# Integrative physiological, transcriptomic, and metabolomic analysis of *Abelmoschus manihot* in response to Cd toxicity

**DOI:** 10.3389/fpls.2024.1389207

**Published:** 2024-06-10

**Authors:** Mengxi Wu, Qian Xu, Tingting Tang, Xia Li, Yuanzhi Pan

**Affiliations:** ^1^ College of Landscape Architecture, Sichuan Agricultural University, Chengdu, China; ^2^ College of Forestry, Sichuan Agricultural University, Chengdu, Sichuan, China

**Keywords:** transcriptomic, metabolomics, Cd stress, lipids, phenylpropanoid, *Abelmoschus manihot*

## Abstract

Rapid industrialization and urbanization have caused severe soil contamination with cadmium (Cd) necessitating effective remediation strategies. Phytoremediation is a widely adopted technology for remediating Cd-contaminated soil. Previous studies have shown that *Abelmoschus manihot* has a high Cd accumulation capacity and tolerance indicating its potential for Cd soil remediation. However, the mechanisms underlying its response to Cd stress remain unclear. In this study, physiological, transcriptomic, and metabolomic analyses were conducted to explore the response of *A. manihot* roots to Cd stress at different time points. The results revealed that Cd stress significantly increased malondialdehyde (MDA) levels in *A. manihot*, which simultaneously activated its antioxidant defense system, enhancing the activities of superoxide dismutase (SOD), peroxidase (POD), and catalase (CAT) by 19.73%–50%, 22.87%–38.89%, and 32.31%–45.40% at 12 h, 36 h, 72 h, and 7 days, respectively, compared with those in the control (CK). Moreover, transcriptomic and metabolomic analyses revealed 245, 5,708, 9,834, and 2,323 differentially expressed genes (DEGs), along with 66, 62, 156, and 90 differentially expressed metabolites (DEMs) at 12 h, 36 h, 72 h, and 7 days, respectively. Through weighted gene coexpression network analysis (WGCNA) of physiological indicators and transcript expression, eight hub genes involved in phenylpropanoid biosynthesis, signal transduction, and metal transport were identified. In addition, integrative analyses of metabolomic and transcriptomic data highlighted the activation of lipid metabolism and phenylpropanoid biosynthesis pathways under Cd stress suggesting that these pathways play crucial roles in the detoxification process and in enhancing Cd tolerance in *A. manihot*. This comprehensive study provides detailed insights into the response mechanisms of *A. manihot* to Cd toxicity.

## 1 Introduction

Cadmium (Cd) is widely recognized as one of the most toxic heavy metals, and the rapid development of modern industry and agriculture has contributed to the increasing prevalence of Cd pollution (Balali-Mood et al., 2021; Rezapour et al., 2022). Importantly, Cd accumulation in soil can easily be transferred to plants and subsequently enter the human food chain posing significant threats to both the soil environment and human health even at low concentrations (Liang et al., 2018). Therefore, it is crucial to implement effective strategies for remediating Cd-contaminated soil. Over the past decade, a variety of strategies, including physical, chemical, and biological methods, have been utilized to decontaminate soil contaminated with Cd. Among these methods, phytoremediation has emerged as a cost-effective and environmentally friendly approach for removing Cd contaminants from soil (Mahar et al., 2016; Suman et al., 2018).

In plants, Cd toxicity either directly or indirectly disrupts various physiological processes, including photosynthesis, respiration, nutrient uptake, and hormonal balance, ultimately resulting in growth retardation, leaf chlorosis, and reduced biomass (Mwamba et al., 2020; Hamid et al., 2022; Nazir et al., 2022). Unlike normal plants, Cd hyperaccumulators are capable of maintaining normal physiological functions in high-Cd environments and accumulate significant amounts of Cd in their tissues (Corso et al., 2018; Haider et al., 2021). Therefore, hyperaccumulators are considered ideal materials for phytoremediation. Identifying new Cd hyperaccumulators and understanding the mechanisms underlying Cd detoxification and accumulation in these plants are crucial for improving the efficiency of phytoremediation (Suman et al., 2018; Kanwar et al., 2020; Yan et al., 2020). Under Cd stress, hyperaccumulators demonstrate an exceptional ability to accumulate Cd in their tissues attributed to the evolution of detoxification mechanisms that mitigate its toxic effects. For instance, in the Cd hyperaccumulator *Solanum nigrum*, Cd binding to sulfur ligands serves as a detoxification mechanism, which likely involves the sequestration of Cd complexes with glutathione or phytochelatins in plant vacuoles leading to greater Cd accumulation than in the nonaccumulator *Solanum melongena* (Pons et al., 2021). Moreover, the cell wall biosynthesis pathway has also been identified as another significant contributor to Cd detoxification in *S. nigrum* (Wang et al., 2022). Similarly, root cell wall modification serves as an important defense strategy for the Cd hyperaccumulator *Sedum alfredii* against Cd stress (Guo et al., 2021). The primary mechanism by which the Cd hyperaccumulator *Erigeron annuus* alleviates Cd toxicity is through the acceleration of antioxidation mechanisms facilitating the removal of reactive oxygen species (ROS) (Zhang et al., 2021). In addition, various metal transporter families, including the ATP-binding cassette transporter (ABC) family, heavy metal ATPase (HMA) family, zinc transporter (ZIP) family, and natural resistance-As-associated macrophage proteins (NRAMPs), have been identified to play crucial roles in the detoxification and accumulation mechanisms of hyperaccumulators (Liu et al., 2017; Zhang et al., 2020). For example, SpHMA3, which is isolated from the Cd/zinc (Zn) hyperaccumulator *Sedum plumbizincicola*, has been demonstrated to play an essential role in Cd detoxification by sequestering Cd into vacuoles in young leaves and stems (Liu et al., 2017). SaNramp1, a plasma membrane-localized transporter, is involved in Cd accumulation in *S. alfredii* (Zhang et al., 2020). However, the majority of proposed Cd detoxification and accumulation mechanisms have focused on specific plants or even specific genotypes. Therefore, expanding the scope of information on the genetics, proteins, and biochemistry of other hyperaccumulators in response to Cd stress is necessary.

*Abelmoschus manihot* (**Supplementary Figure S1**) has been identified as a potential Cd hyperaccumulator that has high ornamental and economic value (Wu et al., 2018). However, the mechanisms underlying the response of *A. manihot* to Cd toxicity remain unknown. Recently, high-throughput omics technologies, including transcriptomics, proteomics, and metabolomics, have been widely applied to study the responses of hyperaccumulators, such as *Phytolacca americana* (Zhao et al., 2011), *S. nigrum* (Wang et al., 2022), *S. alfredii* (Wu et al., 2020), and *Brassica napus* (Zhang et al., 2019), to heavy metal stress. These omics technologies have further expanded our understanding of the complex biological processes induced by Cd stress (Wang et al., 2022; Wei et al., 2023). Therefore, in the present study, we employed a combination of physiological, transcriptomic, and metabolomic analyses to investigate how *A. manihot* acclimates to Cd stress. The objectives of this study were (1) to investigate the physiological response of *A. manihot* to Cd exposure in its roots, (2) to reveal the dynamic adjustments in transcriptional and metabolic processes in *A. manihot* roots in response to Cd stress, and (3) to explore a potential regulatory network between genes and metabolites in *A. manihot* under Cd stress. The results will contribute to a better understanding of the Cd detoxification and accumulation mechanisms of *A. manihot* and further facilitate the decontamination of Cd-contaminated soils.

## 2 Materials and methods

### 2.1 Plant materials and treatments

The seeds of *A. manihot*, sourced from a noncontaminated area in Sichuan Province, China, were subjected to surface sterilization by soaking in 0.05% sodium hypochlorite (NaClO) for 30 min followed by rinsing with deionized water. The sterilized seeds were directly planted in baskets filled with sterilized ceramsite stones and placed within the planting basket of an automatic hydroponic culture device (**Supplementary Figure S2**). Germination occurred under controlled conditions maintaining a constant temperature of 25°C and a photoperiod of 14/10 h (day/night). After 2 weeks, the seedlings were precultured in a greenhouse using Hoagland nutrient solution, with a temperature setting of 28°C/24°C (day/night), a photoperiod of 14/10 h (day/night), and humidity maintained between 60% and 80%. The components of the nutrient solution were as follows: 4 mM Ca(NO_3_)_2_·4H_2_O, 4 mM (NH_4_)_2_SO_4_, 4 mM K_2_SO_4_, 4 mM KNO_3_, 1.3 mM KH_2_PO_4_, 1 mM MgSO_4_·7H_2_O, 50 μM Fe-EDTA, 10 μM H_3_BO_3_, 5 μM MnSO_4_·H_2_O, 5 μM ZnSO_4_·7H_2_O, 1 μM CuSO_4_·5H_2_O, and 0.5 μM Na_2_MoO_4_·2H_2_O (Mwamba et al., 2016). After 30 days of growth, the plants were transferred to Hoagland nutrient solution containing CdCl_2_, while the control plants were not treated with CdCl_2_ (CK). An additional preliminary experiment assessed the physiological parameters of *A. manihot* roots under a range of Cd concentrations, including 0, 50, 100, 200, and 400 μM (data not shown), which allowed the level of Cd exposure to be set at 100 μM in the present study. Plant samples were collected at four different time points: 12 h, 36 h, 72 h, and 7 days. At each time point, a total of 12 individuals were subdivided into three biological replicates. The harvested plants were washed with tap water, and the roots were treated with 20 mM ethylenediaminetetraacetic acid disodium salt (Na_2_-EDTA) for 15 min to remove the Cd adsorbed on the root surface.

### 2.2 Determination of Cd concentrations in plant tissues

Dried samples of roots, stems, and leaves were ground, passed through a 0.15-mm mesh sieve, and digested using an acid mixture of nitric acid (HNO_3_) and perchloric acid (HClO_4_) (v:v, 4:1) (Wu et al., 2018). The Cd concentrations in the different plant tissues were analyzed by atomic absorption spectrometry (PinAAcle 900 H, Perkin Elmer, USA). Electrodeless discharge lamp at 228.8 nm (with a slit width of 0.7 nm) was used as the radiation source for the Cd.

### 2.3 Biochemical parameters

Fresh root tissues were utilized directly for the detection of biochemical parameters. The malondialdehyde (MDA) content was measured using the thiobarbituric acid (TBA) reaction method (Peever and Higgins, 1989). The activities of superoxide dismutase (SOD), peroxidase (POD), and catalase (CAT) were determined following the procedure described by Wu et al. (2018). SOD activity was determined by nitroblue tetrazolium (NBT) reduction, POD activity through guaiacol oxidation, and CAT activity by measuring the decrease in H_2_O_2_ concentration.

### 2.4 Transcriptomic analysis

#### 2.4.1 RNA extraction and transcriptome sequencing

Fresh roots harvested from both Cd-treated and CK samples at different time points were immediately frozen in liquid nitrogen and stored at −80°C for future analysis. Total RNA was extracted using a Plant RNA Purification Kit (Omega, USA). Subsequently, the quantity and quality of total RNA were assessed using spectrophotometry (NanoDrop 2000, Thermo Scientific) and RNase-free agarose gel electrophoresis, respectively. Briefly, mRNAs were enriched from total RNA using oligo(dT)-rich magnetic beads and randomly fragmented using fragmentation buffer. Then, first-strand cDNAs were synthesized with random hexamer primers and reverse transcriptase using mRNA fragments as templates followed by second-strand cDNA synthesis using dNTPs, RNase H, and DNA polymerase I. The resulting cDNA fragments were purified using a QiaQuick PCR extraction kit (Qiagen, Duesseldorf, Germany), end-repaired, poly(A) tailed, and ligated to Illumina sequencing adapters. The ligation products were size selected via agarose gel electrophoresis, PCR amplified, and sequenced on the Illumina HiSeq™4000 platform by Gene Denovo Biotechnology Co., Ltd. (Guangzhou, China) (Grabherr et al., 2011).

Before assembly, the raw reads were filtered by removing low-quality reads containing more than 40% low-quality bases (Q value ≤ 10 bases), adaptor-contaminated reads, and reads with more than 10% unknown bases. The high-quality clean reads were subsequently *de novo* assembled using the Trinity package to construct unique consensus sequences as reference sequences (Grabherr et al., 2011). The assembled unigenes were aligned using the BLASTx program with an E-value ≤ 10^−5^ to various protein databases, including the Nonredundant Protein Sequence Database (Nr, ftp://ftp.ncbi.nih.gov/blast/db/), Swissprot (http://www.uniprot.org/), Kyoto Encyclopedia of Genes and Genomes (KEGG, http://www.genome.jp/kegg/), Gene Ontology (GO, http://geneontology.org/), and Eukaryotic Ortholog Groups (KOG, http://www.ncbi.nlm.nih.gov/KOG/).

The expression levels of unigenes were calculated using RPKM (Reads Per Kilobase of exon Model per Million mapped reads), based on the number of uniquely mapped reads, to eliminate the influence of unigene length and sequencing discrepancies on the expression calculation. The longest transcript was selected for genes with more than one alternative transcript to calculate the RPKM. The differentially expressed genes (DEGs) between the Cd treatment and CK groups at different time points were identified by the DESeq R package with a false discovery rate (FDR) ≤ 0.05 and a |log2(fold change)| ≥ 1. GO enrichment analysis was implemented to assess the functions of DEGs using the GOseq R package, and GO terms with a corrected p ≤ 0.05 were considered significantly enriched. All RNA-seq read data reported in this study have been deposited in the NCBI under project accession number PRJNA1078221.

#### 2.4.2 Gene expression pattern analysis

Short Time-series Expression Miner (STEM) software was used to cluster all DEGs resulting from pairwise comparisons between the Cd treatment and CK groups at four time points (Ernst and Bar-Joseph, 2006). Only expression profiles with p ≤ 0.05 were considered significant temporal expression profiles. Subsequently, GO enrichment analysis was performed on the major expression profiles.

#### 2.4.3 Gene coexpression network analysis

A coexpression network for genes was constructed using the weighted gene coexpression network analysis (WGCNA, v1.47) package in R (Langfelder and Horvath, 2008). After filtering out genes that were not expressed in more than half of the samples, a total of 24,689 genes were selected and imported into WGCNA to construct coexpression modules using the automatic network construction function (blockwise modules). The modules were generated with default settings except for adjustments made to the power (set to 7), TOMType (set to unsigned), and minModuleSize (set to 50). To identify biologically significant modules, module eigengenes were used to calculate correlation coefficients with physiological traits. The networks were visualized using Cytoscape_3.9.1.

#### 2.4.4 Quantitative real-time PCR

Twelve DEGs were randomly selected for qRT−PCR assays to validate the RNA sequencing (RNA−seq) results. Primers were designed using the Primer3 website and are listed in **Supplementary Table 1**. *Actin* was selected as the reference control gene. The PCR system (10 µl) consisted of 1 μl of template cDNA, 0.5 µl of each forward and reverse primer (4 µM), 5 µl of 2× SYBR^®^ Green Supermix, and 3 µl of ddH_2_O. The reaction program consisted of an initial denaturation step at 95°C for 3 min followed by 40 cycles of 95°C for 10 s and 60°C for 30 s. Melting curves were generated from 65°C to 95°C with increments of 1°C for 4 s. Three technical replicates were performed for each sample, and transcript levels were calculated using the 2^−ΔΔCT^ method. Correlation analysis was conducted between the qPCR and RNA-seq results.

### 2.5 Metabolomic analysis

#### 2.5.1 Metabolite extraction and liquid chromatography with mass spectrometry metabolome analysis

The samples from the Cd-treated and CK groups at different time points were retrieved from storage at −80°C, vacuum freeze dried, and ground into a powder. For each sample, approximately 100 mg of the ground material was extracted overnight at 4°C with 1.0 ml of 70% methanol containing 0.1 mg·L^−1^ lidocaine as the internal standard. After centrifugation at 10,000 × *g* for 10 min at 4°C, the supernatants were collected, filtered, and transferred to an injection bottle for liquid chromatography with tandem mass spectrometry (LC−MS/MS) analysis (UPLC, Shim-pack UFLC Shimadzu CBM20A system, MS/MS, Applied Biosystems 4500 QTRAP). Quality control (QC) samples were prepared by mixing 20 μl from each sample to monitor deviations in the analytical results and ensure system stability throughout the entire experiment. The UPLC conditions, as well as the mass spectrometry parameters, were set up as described previously (Chen et al., 2013).

#### 2.5.2 Metabolomic data analysis

The qualitative analysis of metabolites was performed by referencing existing mass spectrometry databases, including MassBank (http://www.massbank.jp/), HMDB (http://www.hmdb.ca/), MoToDB (http://www.ab.wur.nl/moto/), KNAPSAcK (http://kanaya.naist.jp/KNApSAcK/), METLIN (http://metlin.scripps.edu/index.php), and the commercial database MWDB (MetWare Biological Science and Technology Co., Ltd., Wuhan, China) (Zhu et al., 2018). For the quantitative analysis of metabolites, the multiple reaction monitoring (MRM) mode of triple quadrupole mass spectrometry was used (Chen et al., 2013). After obtaining the metabolite spectra for each sample, the areas of the mass spectrum peaks were integrated, and the mass spectra of the same metabolites in different samples were corrected.

Principal component analysis (PCA) and orthogonal partial least squares discriminant analysis (OPLS-DA) were performed using the R package ropls. The model’s stability was assessed through seven cycles of interactive verification. Additionally, Student’s t-test was used. Significantly differentially expressed metabolites (DEMs) between the Cd treatment and CK groups were selected based on the variable weight value (VIP) from the OPLS-DA model and the p-value from Student’s t-test. The metabolites with VIP > 0.1 and p < 0.05 were considered significantly different metabolites (Hu et al., 2020). These DEMs were then subjected to metabolic pathway analysis.

### 2.6 Integrated analysis of the metabolomic and transcriptomic data

The transcriptome and metabolome data were normalized and subjected to statistical analysis to elucidate the relationships between the genes and metabolites implicated in the Cd stress response. Pearson correlation analysis between DEGs and DEMs was performed using the COR function in R with normalized data. Subsequently, DEGs and DEMs with Pearson correlation coefficient (PCC) thresholds of ≥0.8 or ≤−0.8 were subjected to KEGG analysis.

### 2.7 Statistical analysis

Statistical analysis was performed using SPSS 20.0 (SPSS Inc., Chicago, IL, USA). Statistical significance was calculated using two- or one-way ANOVA followed by Tukey’s multiple comparison test with differences deemed significant at p < 0.05. Graphs were generated using GraphPad Prism 9 software (GraphPad Software Inc., La Jolla, CA, USA).

## 3 Results

### 3.1 Cd concentrations in different tissues

**Figure 1:**
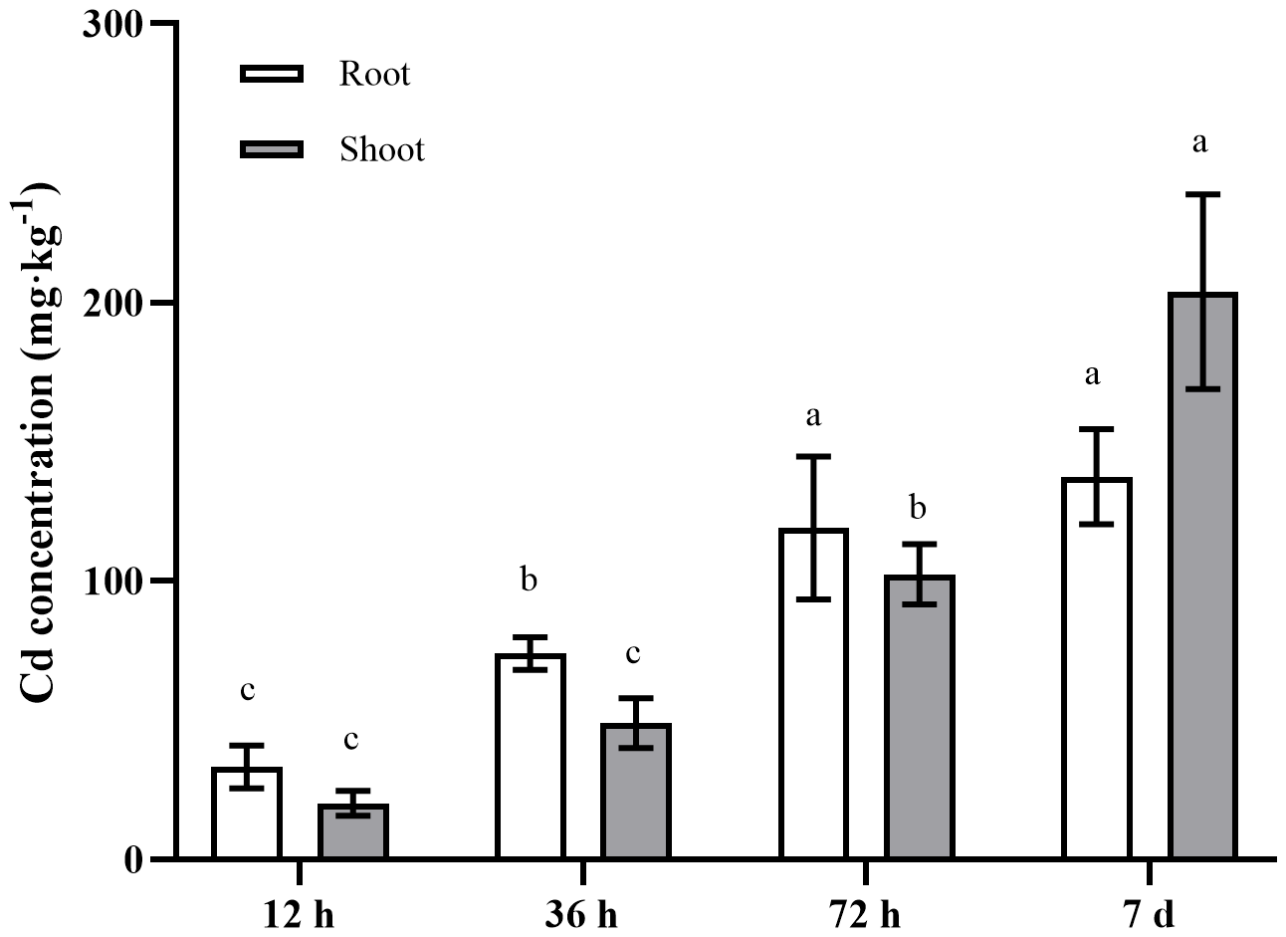
Cd concentrations in different tissues of *A. manihot* at 12 h, 36 h, 72 h, and 7 days. Each error bar represents the mean ± SD. Different small letters indicate significant differences at the p < 0.05 level among different time points in the same tissue.

The present study analyzed the Cd concentrations in the roots, stems, and leaves of *A. manihot* at four different time points following exposure to 100 μM Cd. The results revealed a significant increase in the Cd content in *A. manihot* with increasing duration of Cd exposure (**Figure 1**). The average Cd concentrations in the roots were 33.21, 73.95, 119.00, and 137.38 mg·kg^−1^ at 12 h, 36 h, 72 h, and 7 days after Cd treatment, respectively. Initially, the Cd concentrations in the shoots remained lower than those in the roots at 12–36 h. However, the Cd concentration in the shoots significantly increased to 203.81 mg·kg^−1^ at 7 days indicating a 1.48-fold increase over that in the roots. These results verified the high Cd accumulation capability of *A. manihot*, as indicated by a previous study (Wu et al., 2018).

### 3.2 Effect of Cd stress on physiological characteristics

**Figure 2:**
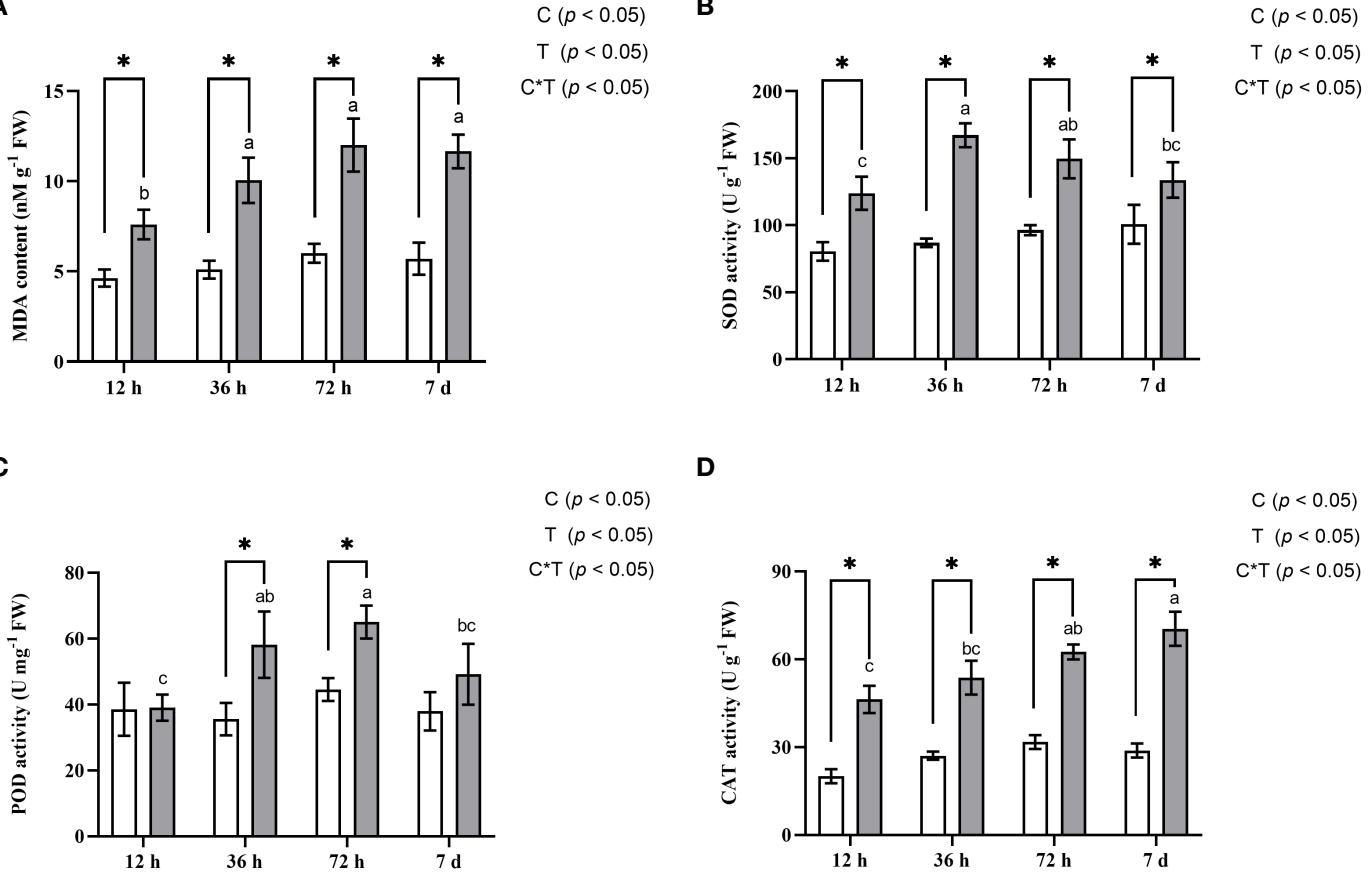
Effects of Cd stress on the content of MDA **(A)** and the activities of SOD **(B)**, POD **(C)**, and CAT **(D)** in the roots of *A*. *manihot* at four time points. The white columns represent the CK group, and the gray columns represent the Cd treatment group. Each error bar represents the mean ± SD. Different lowercase letters denote statistically significant differences among the various Cd stress durations (Duncan’s test, p < 0.05). The symbol “*” indicates a significant difference (t-test, p < 0.05) between the Cd and CK treatments at the corresponding sampling time. Two-way ANOVA results for treatment (T) and treatment time (T) are shown in the insets.

*A. manihot* demonstrated strong tolerance to Cd treatment showing no visible phytotoxicity symptoms. To evaluate the response of *A. manihot* roots to Cd stress, several biochemical indices associated with the oxidative stress response were monitored. MDA, one of the final products of cell membrane lipid peroxidation, is recognized as a well-known biomarker for oxidative lipid damage caused by increased ROS under abiotic stress (Wu et al., 2018; Yu et al., 2021). Compared with that in the control plants, the MDA content in the Cd-treated plants significantly increased by 64.0%–104.12% indicating that Cd induced cellular oxidative stress (**Figure 2A**). Plants respond to oxidative stress by enhancing their antioxidant defense systems (Wu et al., 2018; Zhang et al., 2021). In the roots of *A. manihot*, compared with those in the CK treatment, Cd stress significantly increased the activity of SOD, POD, and CAT by 19.73%–50%, 22.87%–38.89%, and 32.31%–45.40%, respectively, at 12 h, 36 h, 72 h, and 7 days. SOD activity significantly increased under Cd stress, peaking at 36 h, and then gradually decreased (**Figure 2B**). A similar pattern was observed for POD activity, which peaked at 72 h (**Figure 2C**). Additionally, the CAT activity in roots increased continuously with the duration of Cd exposure (**Figure 2D**).

### 3.3 Transcriptome analysis of *A. manihot* roots under Cd stress

After data filtering, approximately 40.61–66.83 million 150-bp high-quality clean reads were obtained from the RNA-seq data of 24 samples. The clean read Q20 and Q30 values for all test samples were greater than 97.51% and 92.74%, respectively (**Supplementary Table S2**) demonstrating the high quality of sequencing and the feasibility of subsequent analyses. *De novo* assembly of the clean reads using the Trinity program generated a reference transcriptome comprising 73,122 unigenes, with an average length of 873 bp, a maximum length of 13,204 bp, a minimum length of 201 bp, and an N50 of 1,268 bp. Using the BLASTX tool with a cutoff E-value of 10^−5^, a total of 56,752 unigenes were functionally annotated (**Supplementary Table S3**). Based on extensive database searches, *Gossypium hirsutum* (22.47%), *Gossypium arboreum* (22.14%), and *Gossypium raimondii* (21.91%) exhibited high similarity to *A. manihot* (**Supplementary Figure S3**).

To further evaluate the validity of the RNA-seq data, 12 unigenes were randomly selected for qPCR analysis. The RT-qPCR results for these genes showed similar expression trends to the RNA-seq data (**Supplementary Figure S4**) indicating the accuracy of the RNA-seq data. Furthermore, a strong positive correlation was observed between the qPCR and RNA-seq data (r = 0.7581, p < 0.001) (**Supplementary Figure S5**). These results confirm the validity of RNA-seq and suggest that the RNA-seq data can be reliably used for further analyses.

### 3.4 Analysis of the DEGs in *A. manihot* at different time points under Cd stress

**Figure 3:**
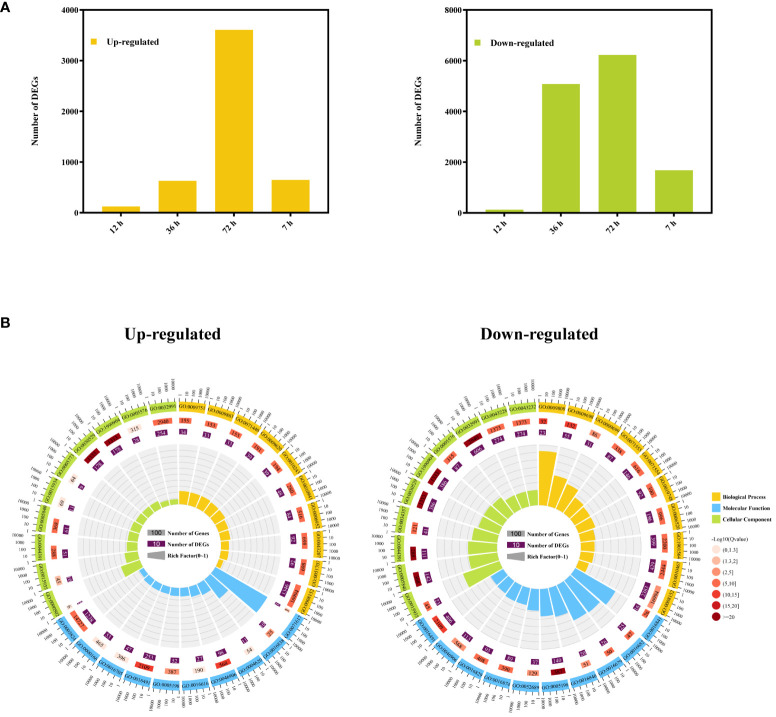
Cd-responsive differentially expressed genes at different time points. **(A)** The numbers of Cd-responsive DEGs in the roots of *A*. *manihot* at four time points. **(B)** GO enrichment circle diagram of upregulated and downregulated DEGs. The first 10 enrichment cycles of GO terms are depicted, and the number of genes is presented outside the circle on a logarithmic scale. Different colors represent different GO domains. The second circle displays the number of genes enriched in each GO term along with the Q value. The length of the bar corresponds to the number of genes, with a shorter bar and a redder color indicating a lower Q value. The third circle represents the number of DEGs enriched in each GO term. The fourth circle shows the enrichment factor value for each GO term, with each grid line representing 0.1 in the background.

The DEGs of *A. manihot* were identified by comparing RNA-seq data from Cd-treated root samples with CK samples at the same time points. Comparative DEG analysis revealed a total of 245 DEGs (122 upregulated and 123 downregulated) at 12 h, 5,708 DEGs (629 upregulated and 5,079 downregulated) at 36 h, 9,834 DEGs (3,606 upregulated and 6,228 downregulated) at 72 h, and 2,323 DEGs (647 upregulated and 1,676 downregulated) at 7 days (**Figure 3A**).

Subsequently, the functions of the DEGs were classified according to GO classifications. The downregulated DEGs were predominantly associated with “structural molecule activity” in the molecular function category, “intracellular ribonucleoprotein complex” and “ribonucleoprotein complex” in the cellular component category, and “phenylpropanoid metabolic process,” “lignin metabolic process,” and “cell wall organization or biogenesis” in the biological process category (**Figure 3B**, **Supplementary Table S4**). These results suggest that Cd stress impacts the cell wall organization or biogenesis of *A. manihot*. In addition, the upregulated DEGs were significantly enriched in molecular function categories, such as “tetrapyrrole binding,” “catalytic activity,” and “oxidoreductase activity,” while the cellular component category was dominated by “intracellular ribonucleoprotein complex,” “ribonucleoprotein complex,” and “ribosome.” In addition, the biological process category was the most enriched in “defense response,” “metabolic process,” and “salicylic acid-mediated signaling pathway” (**Figure 3B**, **Supplementary Table S4**). These results indicate that *A. manihot* can enhance catalytic activity and tetrapyrrole binding and activate defense mechanisms and salicylic acid-mediated signaling pathways to achieve stable Cd detoxification or improve Cd tolerance (Yan et al., 2020).

### 3.5 Temporal trends in DEGs responding to Cd stress

**Figure 4:**
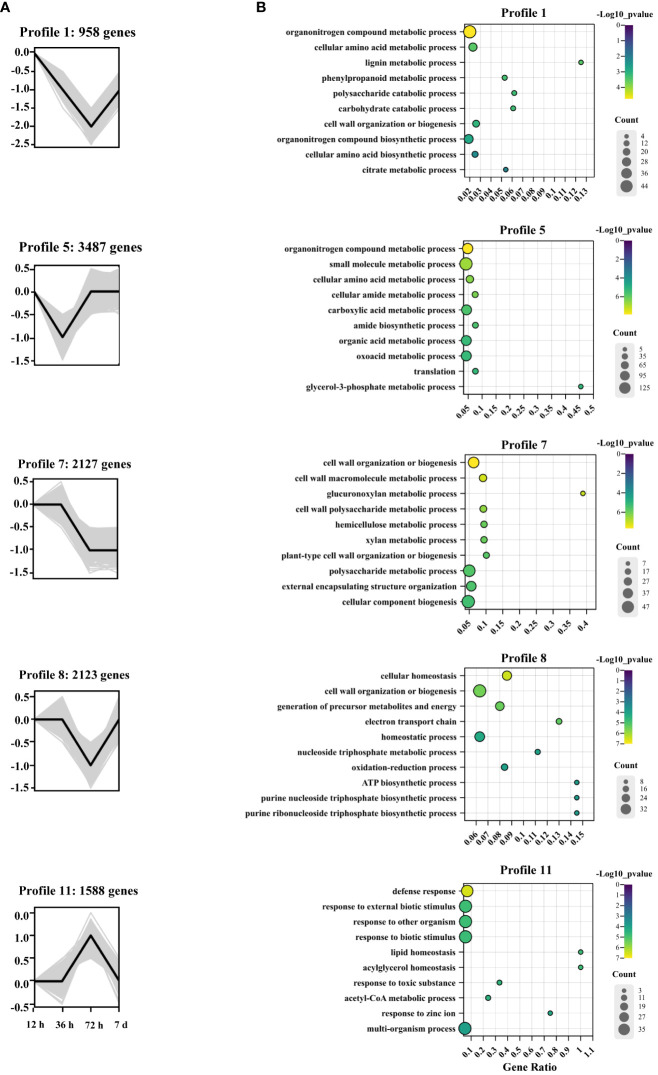
Patterns of gene expression and GO enrichment across four time points in the roots of *A*. *manihot*. **(A)** Expression profiles of five significant DEGs (*p* < 0.05). In each frame, the black lines represent the expression tendencies of genes. The number of genes belonging to each pattern is labeled above the frame. **(B)** GO enrichment analysis of significant gene expression profiles. The GO analysis diagram was generated using ChiPlot (https://www.chiplot.online/). The significance of the most represented GO-slims in each profile is indicated by the p-value. The top 10 significant pathways according to the biological process results are displayed.

To analyze the temporal expression patterns of Cd-responsive genes, STEM analysis was employed to cluster DEGs with similar expression patterns (**Supplementary Figure S6**). In the STEM analysis, profiles 1, 5, 7, 8, and 11 with p < 0.05 were considered significant profiles (**Figure 4A**). Subsequently, GO enrichment analysis of the DEGs within these significant profiles was performed to elucidate the functional significance of the transcriptional changes induced by Cd stress (**Figure 4B**). In profiles 1 and 7, genes associated with cell wall biosynthetic and metabolic pathways were particularly enriched, including “glucuronoxylan metabolic process,” “xylan metabolic process,” “lignin metabolic process,” “phenylpropanoid metabolic process,” and “polysaccharide catabolic process.” Gene expression in profile 7 decreased after 36 h, whereas in profile 1, there was a decrease in gene expression from 12 to 72 h, which subsequently increased after 72 h. Profiles 5 and 8 also shared a similar expression pattern to profile 1, where genes were primarily associated with energy and substance metabolism. Profile 5, enriched in GO terms related to “glycerol-3-phosphate metabolic process,” showed a decrease in gene expression levels from 12 to 36 h followed by an increase thereafter. Profile 8, enriched in GO terms, such as “ATP biosynthetic process,” “purine nucleoside triphosphate biosynthetic process,” and “purine ribonucleoside triphosphate biosynthetic process,” demonstrated a decrease in gene expression from 36 to 72 h but an increase afterward. In contrast, profile 11 exhibited genes that were upregulated between 36 and 72 h and subsequently downregulated after 72 h. The enriched pathways represented in this profile included “lipid homeostasis,” “acylglycerol homeostasis,” “response to toxic substances,” and “response to zinc ion” (**Figure 4B**).

### 3.6 Coexpression network analysis and identification of hub genes related to the response to Cd stress

**Figure 5:**
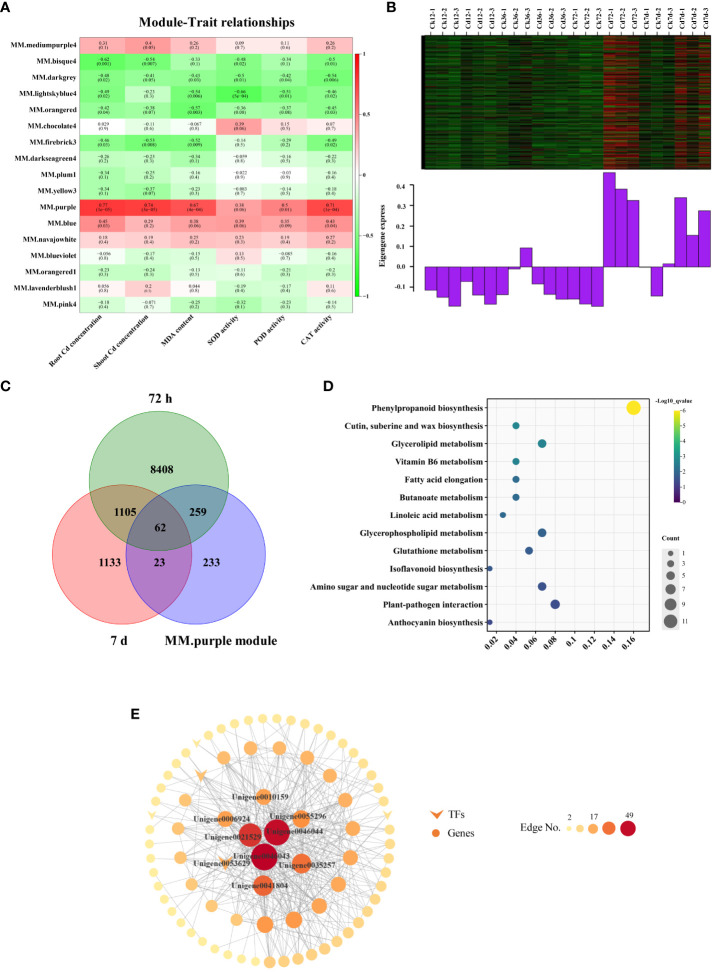
Module–trait relationships based on WGCNA. **(A)** Heatmap of the correlation coefficients between WGCNA modules and traits. Each row represents a specific module, and each column represents a trait. The numbers in each cell indicate the correlation coefficients and the corresponding p-values (in parentheses). **(B)** Gene coexpression heatmap for the purple module (upper panel) and the expression level of the corresponding eigengene in each sample (lower panel). **(C)** Common genes between DEGs (72 h and 7 days) and the purple module. **(D)** KEGG analyses of the overlapping DEGs. **(E)** The correlation network of the purple module. Genes with edge weights >0.17 were visualized by Cytoscape. The size and color of each node represent the number of connections.

WGCNA was used to investigate various physiological parameters and key genes involved in Cd detoxification and accumulation in *A. manihot*. This analysis identified 17 modules with an unsigned TOM through the dynamic tree cutting (**Supplementary Figure S7**). Notably, the purple module exhibited significant positive correlations with Cd accumulation in both roots (r = 0.77, p < 0.05) and shoots (r = 0.74, p < 0.05), as did MDA (r = 0.67, p < 0.05), POD (r = 0.50, p < 0.05), and CAT (r = 0.71, p < 0.05) (**Figure 5A**). We speculated that genes in the purple module were associated with enhanced resistance and Cd accumulation in *A. manihot*. Therefore, the purple module was selected for further analysis.

There were 577 genes in the purple module. Based on the gene expression heatmaps and eigengene histograms, the genes within the purple module were upregulated at 72 h and 7 days following Cd treatment (**Figure 5B**). Among them, 321 and 85 genes in the purple module were differentially expressed at 72 h and 7 days, respectively (**Figure 5C**). Subsequently, KEGG pathway analysis was conducted on the overlapping DEGs (344 genes in total). The overlapping DEGs predominantly mapped to pathways, such as “phenylpropanoid biosynthesis,” “glutathione metabolism,” “vitamin B6 metabolism,” and various lipid metabolism-related pathways, including “glycerolipid metabolism,” “glycerophospholipid metabolism,” “fatty acid elongation,” and “linoleic acid metabolism” (**Figure 5D**).

In the purple module, the eight genes with the most connections in the network were considered hub genes (**Figure 5E**, **Supplementary Table S5**). Among these genes, four hub genes were involved in the phenylpropanoid biosynthesis pathway, including three *POD* homologs (*Unigene0046043*, *Unigene0046044*, and *Unigene0010159*) and a *coniferyl-aldehyde dehydrogenase* (*REF1*) homolog (*Unigene0006924*). Additionally, *Unigene0053629* is a homolog of the ethylene-responsive transcription factor ERF98, which belongs to the AP2/ERF transcription factor family. Other hub genes included an ABC transporter homolog (*Unigene0055296*), a cytochrome P450 CYP82D47 homolog (*Unigene0041804*), and a RING-H2 finger protein ATL3 homolog (*Unigene0035257*). Taken together, these core physiological processes and the hub genes that regulate them may be involved in the regulation of *A. manihot*’s response to Cd in terms of uptake, translocation, and detoxification.

### 3.7 Effects of Cd stress on root metabolism in *A. manihot*

**Figure 6:**
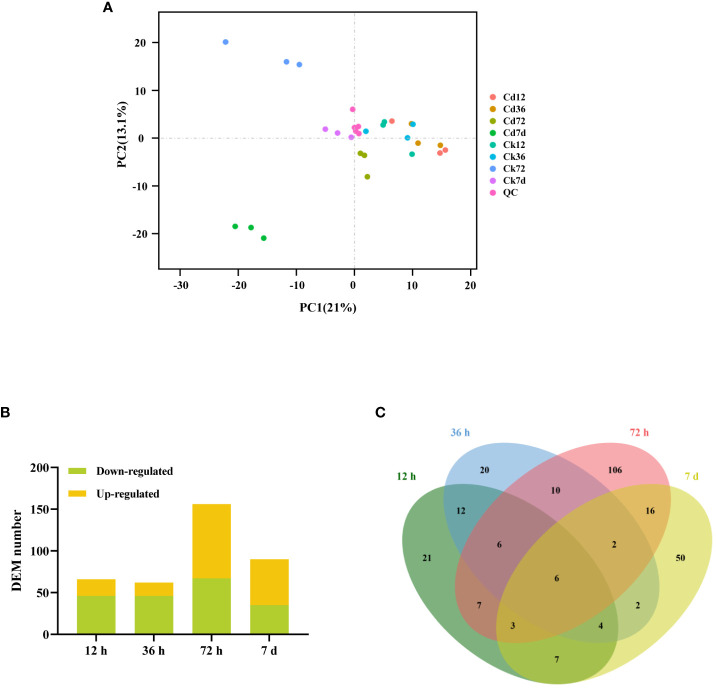
Metabolite accumulation in *A*. *manihot* roots at different time points under Cd stress. **(A)** PCA score plot based on metabolome data, with PC1 and PC2 plotted on the x- and y-axes, respectively. Quality control (QC) samples, prepared from a mixture of sample extracts, were used to ensure the reproducibility of measurements under the same treatment method. **(B)** Total numbers of up- and downregulated DEMs at different time points. **(C)** Venn diagram comparing the DEM numbers in pairwise groups.

To investigate the simultaneous changes in the metabolome and identify the metabolic adaptations of *A. manihot* to Cd stress, a broadly targeted metabolomic analysis of its roots was performed. Using an LC-MS/MS-based widely targeted metabolomics approach, we detected a total of 722 metabolites (**Supplementary Table S6**). Multivariate PCA and OPLS-DA were subsequently applied to identify differentially abundant metabolites across various time points. The PCA results showed that biological repeat samples within each group clustered together demonstrating the stability and reliability of the sequencing results and confirming good reproducibility among samples in each group (**Figure 6A**). Notably, as the duration of Cd exposure increased, distinct differences emerged among the samples. According to predefined criteria, a total of 66 (20 upregulated, 46 downregulated), 62 (16 upregulated, 46 downregulated), 156 (89 upregulated, 67 downregulated), and 90 (55 upregulated, 35 downregulated) DEMs were identified at 12 h, 36 h, 72 h, and 7 days, respectively (**Figure 6B**). These findings highlight the differences in metabolic responses at various stages of Cd stress, suggesting the occurrence of adaptive metabolic adjustments in *A. manihot* as the stress progresses.

Among the identified DEMs, six metabolites consistently appeared across all time points, including D-pipecolinic acid, adenosine O-ribose, melatonin, N,N-didesvenlafaxine, LysoPE 18:3, and LysoPE 18:1 (**Figure 6C**). The upregulated DEMs were categorized into 12 major groups, predominantly including alkaloids, lipids, flavonoids, phenolic acids, organic acids, amino acids, and nucleotide and its derivatives (**Supplementary Table S7**). Furthermore, the analysis revealed the participation of several phytohormones, including abscisic acid and gibberellin, in the plant’s adaptive response to Cd stress (**Supplementary Table S7**, **Supplementary Figure S8**). Further analysis through KEGG pathway analysis of these upregulated DEMs highlighted their involvement in fatty acid-related pathways such as “linoleic acid metabolism,” “α-linolenic acid metabolism,” “fatty acid biosynthesis,” and “fatty acid degradation” (**Supplementary Figure S8**).

### 3.8 Integrated analysis of metabolomic and transcriptomic data

**Figure 7:**
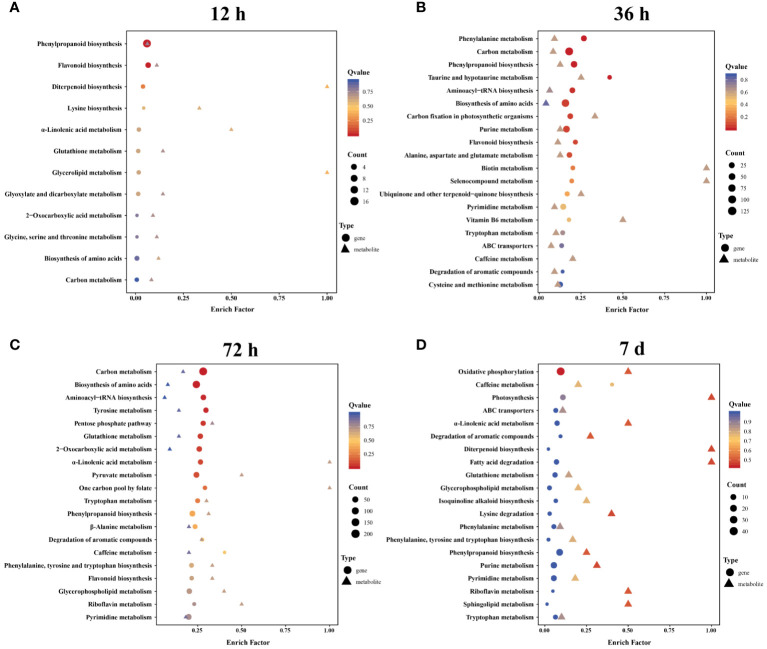
The KEGG enrichment pathways that integrate metabolomic and transcriptomic data at 12 h **(A)**, 36 h **(B)**, 72 h **(C)**, and 7 days **(D)**. The color gradient from red to yellow to blue represents the significance of enrichment, which changes from high to medium to low, as indicated by the Q value.

**Figure 8:**
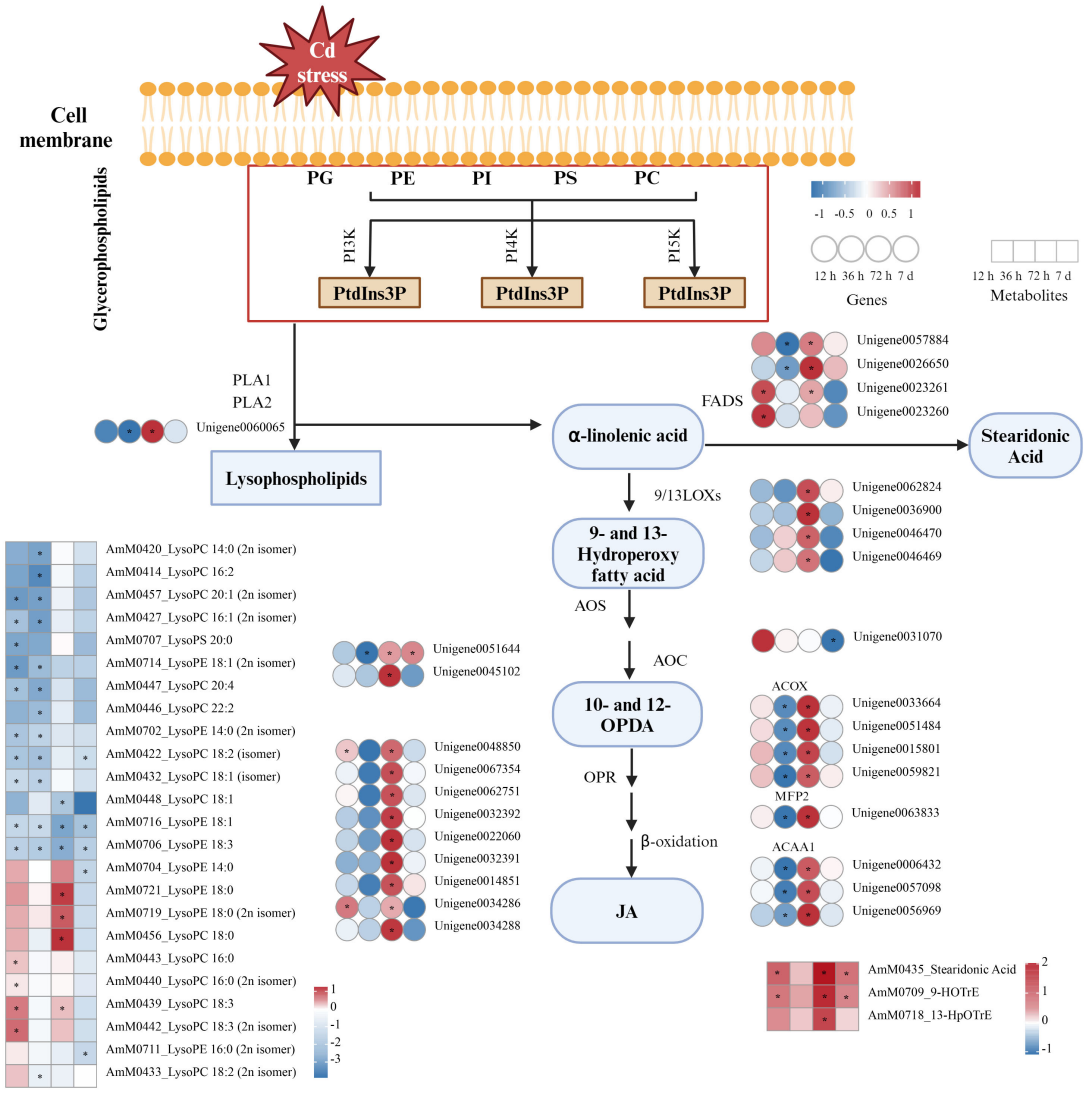
DEGs and DEMs associated with lipid metabolism pathways in *A. manihot* roots in response to Cd stress. The heatmap was constructed using Log_2_FC values. An asterisk “*” denotes a significant difference between the Cd treatment group and the control group (p < 0.05). Different colors represent differential expression, with red indicating high expression and green indicating low expression. PC, phosphatidylcholine; PE, phosphatidylethanolamine; PG, phosphatidylglycerol; PI, phosphatidylinositol; PS, phosphatidylserine; PI3K, PI4K, PI5K, phosphatidylinositol 3-kinase, 4-kinase, and 5-kinase, respectively; PtdIns3P, PtdIns4P, and PtdIns5P, phosphatidylinositol 3-phosphate, 4-phosphate, and 5-phosphate, respectively; PLA, phospholipase A; LOX, lipoxygenase; AOS, allene oxide synthase; AOC, allene oxide cyclase; FAD, fatty acid desaturase; OPR, 12-oxophytodienoate reductase; ACX, acyl-CoA oxidase; MFP2, enoyl-CoA hydratase/3-hydroxyacyl-CoA dehydrogenase; ACCA1, acetyl-CoA acyltransferase; JA, jasmonic acid; 10-OPDA, 10-oxo-11,15-phytodienoic acid; 12-OPDA, 12-oxo-10,15-phytodienoic acid.

**Figure 9:**
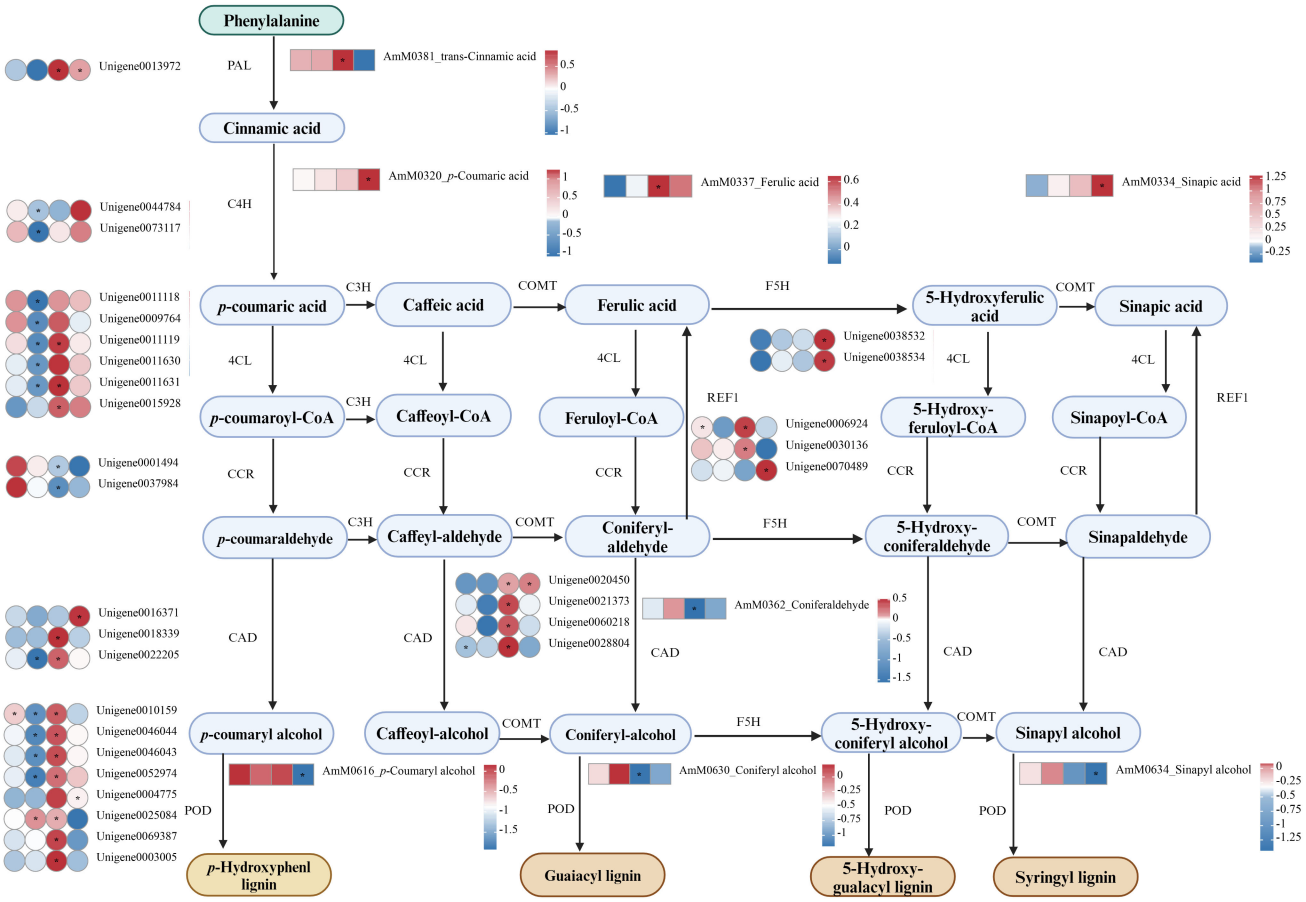
DEGs and DEMs involved in phenylpropanoid biosynthesis pathways in *A. manihot* roots in response to Cd stress. The heatmap was constructed using Log_2_FC values. An asterisk “*” denotes a significant difference between the Cd treatment group and the control group (p < 0.05). Different colors represent differential expression, with red indicating high expression and green indicating low expression. PAL, phenylalanine ammonia lyase; C4H, cinnamate 4-hydroxylase; COMT caffeic acid O-methyltransferase; F5H ferulate 5‐hydroxylase; 4CL, 4-coumarate-CoA ligase; C3H, *p*-coumarate 3-hydroxylase; POD, peroxidase; REF1, coniferyl-aldehyde dehydrogenase.

To gain further insight into the potential regulatory mechanisms influenced by Cd stress, we conducted an integrated analysis of the metabolome and transcriptome data from the roots of *A. manihot*. KEGG enrichment analysis of correlated DEGs and DEMs revealed that phenylpropanoid biosynthesis was significantly enriched in both the metabolome and transcriptome datasets across the four time points (**Figure 7**, **Supplementary Table S8**). Furthermore, lipid metabolism-related pathways, which were enriched at all four time points, exhibited temporal differences. In summary, the analysis of metabolome and transcriptome data identified lipid metabolism, especially α-linolenic acid metabolism, and phenylpropanoid biosynthesis as key metabolic pathways involved in the response of *A. manihot* to Cd stress. Hence, a detailed analysis of the lipid metabolism and phenylpropanoid biosynthesis pathways was conducted (**Figures 8**, **9**).

In the lipid metabolism pathway, significant variations in the levels of lysophospholipids, including lysophosphatidylcholines (LysoPCs), lysophosphatidylethanolamines (LysoPEs), and lysophosphatidylserines (LysoPSs), were observed. At 12 h, there were significant increases in the levels of lysoPC C16:0 (2) and C18:3 (2). Interestingly, most lysoPEs showed significant decreases relative to the controls, except for lysoPE 18:0 (2), which increased at 72 h. Furthermore, *phospholipase A* (*PLA*), a crucial gene involved in the metabolism of fatty acids and lysophospholipids, was significantly downregulated at 36 h and subsequently upregulated at 72 h. At common time points, significant upregulation of metabolites, such as 13-hydroperoxyoctadecatrienoic acid (13-HPOTrE), 9-hydroxy-10E,12Z,15Z-octadecatrienoic acid (9-HOTrE), and stearidonic acid in α-linolenic acid metabolism, was also observed under Cd stress. Consistent with these findings, the reaction products of upstream genes, including *fatty acid desaturase* (*FAD*) and *lipoxygenase* (*LOX*), were significantly upregulated at 72 h in response to Cd stress.

13-HPOTrE serves as the key intermediate in the biosynthesis pathway of jasmonic acid (JA) from the α-linolenic acid metabolic pathway (Feussner and Wasternack, 2002). The sustained upregulation of 13-HPOTrE suggested that the synthesis of JA from α-linolenic acid is strongly induced by Cd stress. Moreover, the expression levels of several JA pathway-related structural genes, including *allene oxide synthase* (*AOS*) and *12-oxophytodienoate reductase* (*OPR*), and β-oxidation-related genes, such as *acyl-CoA oxidase* (*ACX*), *enoyl-CoA hydratase/3-hydroxyacyl-CoA dehydrogenase* (*MFP2*), and *acetyl-CoA acyltransferase* (*ACAA1*), were upregulated at 72 h. These results provide further evidence that the synthesis of JA was strongly induced by Cd stress.

In the phenylpropanoid biosynthesis pathway, Cd stress triggered the upregulation of *phenylalanine ammonia lyase* (*PAL*), *cinnamate 4-hydroxylase* (*C4H*), *caffeic acid O-methyltransferase* (*COMT*), *ferulate 5-hydroxylase* (*F5H*), *4-coumarate-CoA ligase* (*4CL*), and *POD* expression at 72 h or 7 days. This resulted in marked increases in the production of p-coumaric acid, trans-cinnamic acid, ferulic acid, and sinapic acid. In addition, REF1 exhibited upregulated expression at 72 h and 7 days. Concurrently, there were significant decreases in the concentrations of three primary monolignols: sinapyl alcohol, *p*-coumaryl alcohol, and coniferyl alcohol. Given the critical role of POD in the final steps of phenylpropanoid biosynthesis, particularly in lignin polymerization through the oxidation of monolignols, the reductions in these precursors coupled with the upregulation of POD may lead to an increase in lignin content.

## 4 Discussion

Cd, a deleterious nonessential element, has negative effects on plant growth and development (Asgari Lajayer et al., 2017; Haider et al., 2021; Khalid et al., 2022). Despite their high Cd accumulation capacity, many hyperaccumulators exhibit slow growth and low biomass, which limits their effectiveness in remediation (Yan et al., 2020; Wang et al., 2022). Our previous study revealed that concentrations of Cd below 100 mg·kg^−1^ stimulate the growth of *A. manihot* in Cd-polluted soil while maintaining a high translocation factor (Wu et al., 2018). Similarly, in this study, *A. manihot* demonstrated the ability to accumulate high amounts of Cd in aboveground tissues without exhibiting obvious physiological toxicity when exposed to 100 μM Cd. Furthermore, Cd accumulation in the roots and shoots of *A. manihot* significantly increased as the duration of Cd stress increased (**Figure 1**). Given its ability to hyperaccumulate Cd, *A. manihot* has significant application prospects. Therefore, further investigations are necessary to explore the mechanisms underlying the response to Cd stress in *A. manihot*.

### 4.1 Antioxidant enzymes alleviate the toxicity of Cd in *A. manihot*

Exposure to Cd disturbs the redox homeostasis of plants, leading to pronounced increases in ROS production and lipid peroxidation, consequently triggering oxidative stress (Asgari Lajayer et al., 2017; Haider et al., 2021). In response to Cd stress, physiological traits, which are pivotal for plant survival and adaptation under challenging conditions, play significant roles in orchestrating defense strategies (Pan et al., 2021; Xu et al., 2022). A noteworthy example of such physiological responses is the activation and regulation of antioxidant enzymes under Cd stress, which play essential roles in restoring cellular functions and maintaining homeostasis (Yu et al., 2021; Liu et al., 2023). Our findings indicate that Cd stress induced a significant increase in the content of the lipid peroxidation product MDA suggesting increased lipid oxidative damage in plant cells (**Figure 2A**). Moreover, exposure to Cd stress significantly elevated the activity of antioxidant enzymes (SOD, POD, and CAT) in *A. manihot* roots in this study (**Figures 2B–D**), consistent with findings in Cd hyperaccumulators such as *Youngia japonica* (Yu et al., 2021), *Pterocypsela laciniata* (Zhong et al., 2019), and *Arabis paniculate* (Liu et al., 2023). Among these enzymes, POD plays pivotal roles in regulating diverse plant physiological processes under Cd stress (Rui et al., 2016; Loix et al., 2017). In the roots of *A. manihot*, there was no significant difference in POD activity compared to that in the CK group at 12 h, but a significant increase was observed thereafter (**Figure 2C**). This finding aligns with previous research suggesting that POD becomes particularly important in the later stages of exposure to Cd toxicity (Yang et al., 2007; Rui et al., 2016).

### 4.2 *A. manihot* copes with Cd stress by adjusting gene expression

In this study, transcriptome analysis revealed significant changes in the transcripts of *A. manihot* roots from 12 h to 7 days under Cd stress. At the initial stage of Cd stress, only a limited number of genes exhibited differential regulation. Notably, the transcript-level responses of *A. manihot* to Cd stress became more pronounced during the medium term (36–72 h) (**Figure 3A**). Moreover, more downregulated DEGs were identified in *A. manihot* roots than upregulated DEGs under Cd stress at different time points (**Figures 3A** and **6B**). These downregulated DEGs were mainly enriched in metabolic pathways related to cell wall organization or biogenesis (**Figure 3B**, **Supplementary Table S4**). Interestingly, DEGs involved in secondary cell wall synthesis exhibited diverse temporal expression patterns in response to Cd stress. For example, DEGs related to hemicelluloses, such as xylan and glucuronoxylan, decreased after 36 h, while those linked to lignin metabolic processes declined continuously from 12 to 72 h before increasing thereafter (**Figure 4**). The cell wall is a dynamic structure whose composition rapidly changes in response to Cd stress (Loix et al., 2017). In general, elevated levels of polysaccharide components result in greater accumulation of metal ions within the cell wall, thereby impeding their entry into the protoplast (Guo et al., 2021; Wang et al., 2022). This serves as a significant defense mechanism against trace metal stress across many plant species. However, studies on hyperaccumulators have revealed that the fixation of metal ions by root cell walls is less stable than that in non-hyperaccumulator plants potentially leading to the transportation of metal ions to the shoots (Loix et al., 2017; Guo et al., 2020). For example, the Cd non-hyperaccumulating ecotype of *S. alfredii* exhibits abundant functional groups and xyloglucan in hemicellulose resulting in tighter Cd binding within the root cell wall. Conversely, the Cd-hyperaccumulating ecotype of *S. alfredii* shows lower levels of hemicellulose-bound Cd, which allows easier transport into the shoot (Guo et al., 2020). Therefore, during a period of Cd exposure, the dynamic inhibition of the secondary cell wall in *A. manihot* may facilitate the upward transport of Cd ions.

Similarly, we found that other metabolic processes were also dynamically regulated under Cd stress in *A. manihot*. For example, during the middle of Cd exposure, the STEM analysis revealed initial increases followed by decreases in the expression levels of DEGs related to lipid and acylglycerol homeostasis, while DEGs in pathways involved in ATP synthesis exhibited decreased expression followed by increased expression (**Figure 4**). Lipid homeostasis involves a balance between the accumulation of membrane lipids and the accumulation of storage lipids such as triacylglycerol. Previous studies have demonstrated that lipid homeostasis is known to exert systemic effects capable of influencing plant survival, growth, and development during stress (Boutté and Jaillais, 2020). In *A. manihot*, the dynamic control of lipid homeostasis responds to disruptions in lipid metabolism induced by Cd stress. Taken together, these findings demonstrate that Cd stress dynamically affects the gene expression of *A. manihot* in a temporally specific manner.

We utilized physiological indicators as trait files to conduct further analysis of these DEGs. By performing WGCNA, we identified eight hub genes that likely play key roles in Cd stress responses (**Figure 5**, **Supplementary Table S5**). Interestingly, among these hub genes, four were homologous genes related to lignin biosynthesis, including three *POD* genes and one *REF1* homolog. In terms of the antioxidant system, POD assists plants in coping with the excess H_2_O_2_ induced by Cd exposure. In addition to its role in redox homeostasis, this enzyme family participates in various cellular processes, including cell wall loosening, cross-linking, and lignification (Rui et al., 2016; Loix et al., 2017). *POD* is involved in a complex redox network in *A. manihot* playing a regulatory role in response to Cd in terms of accumulation and detoxification. Furthermore, a crucial transcription factor has been identified. *ERF98*, an ethylene response factor, serves as a central regulatory hub in plant responses to abiotic stresses (Wu et al., 2022). Among the identified hub genes, we also identified a homolog of an ABC transporter, a homolog of cytochrome P450 CYP82D47, and a homolog of the RING-H2 finger protein ATL3. ABC transporters, a significant portion of the membrane protein family, play vital roles in the transport and detoxification of heavy metals. In *Arabidopsis*, *AtABCC3* acts as a transporter for PC–Cd complexes influencing Cd accumulation and tolerance (Brunetti et al., 2015). Similarly, *OsABCG36* is activated under heavy metal stress serving as one of the root hub genes responsible for exporting Cd or Cd conjugates, thereby increasing Cd tolerance in *Oryza sativa* (Fu et al., 2019).

### 4.3 Metabolite expression in *A. manihot* is affected by Cd stress

Metabolites represent the biochemical end products of gene activity reflecting the adaptability of organisms (Wu et al., 2023). In this study, we observed a trend of increasing changes in the number of DEMs with prolonged Cd stress peaking at 72 h before decreasing thereafter. (**Figure 6B**). The upregulated DEMs were predominantly composed of alkaloids, lipids, flavonoids, phenolic acids, organic acids, amino acids, nucleotides, and their derivatives (**Supplementary Table S7**). Interestingly, at the early stage of Cd stress, there was a significant increase in the level of abscisic acid potentially triggering intricate stress-adaptive signaling cascades (Shen et al., 2022). Additionally, melatonin was significantly upregulated at all time points (**Figure 6C**) consistent with previous findings (Ahammed et al., 2024). Remarkably, exogenous melatonin application has been demonstrated to enhance Cd tolerance in *Solanum lycopersicum* through increased antioxidant potential and phytochelatin biosynthesis (Hasan et al., 2015). Therefore, the sustained increase in melatonin levels may have contributed to the enhanced tolerance of *A. manihot* to Cd stress. Subsequently, KEGG analysis of the metabolome revealed that the response of *A. manihot* to Cd stress involves various metabolic pathways at different stages (**Supplementary Figure S8**). These upregulated DEMs were mainly enriched in fatty acid-related pathways indicating the importance of fatty acid metabolism in the response of *A. manihot* to Cd stress.

### 4.4 Involvement of lipid metabolism in the adaptive regulation of *A. manihot* under Cd stress

In this study, analysis of time-course metabolome and transcriptome data, along with their integrated results, revealed the crucial role of lipid metabolism-related pathways in the defense response of *A. manihot* to Cd stress (**Figures 4**, **5**, **7**, and **S8**). Similarly, a study on *Tamarix hispida* reported that lipid synthesis and metabolism play key roles in enhancing Cd tolerance (Xie et al., 2023). Lipids, which are fundamental components of plant cell biofilms, participate in a wide range of biological processes, including growth, development, and stress responses (Hou et al., 2016; Hu et al., 2023; Liang et al., 2023). Furthermore, accumulating evidence suggests a role for lipid molecules, such as lysophospholipids, fatty acids, and phosphatidic acid, in plant signaling processes (Liang et al., 2023).

In this study, the levels of various types of lysophospholipids exhibited diverse patterns of change (**Figure 8**). LysoPCs [C16:0 (2) and C18:3 (2)] significantly increased under Cd exposure at 12 h. LysoPCs are typically present in trace quantities in plant tissues, but their levels substantially increase under abiotic or biotic stress conditions (Okazaki and Saito, 2014). Previously, it was reported that the amounts of lysoPCs (C14:0, C15:0, C16, C17:1, and C18:3) significantly increased under cold stress (Sun et al., 2021). At an early stage, the increased generation of lysoPCs in *A. manihot* roots may mediate signaling cascades leading to alterations in gene expression during Cd stress responses (Cappa and Pilon-Smits, 2014). Interestingly, the majority of the detected LysoPEs, specifically LysoPE 18:3 and LysoPE 18:1, decreased consistently at all treatment time points. This result is consistent with findings on *T. hispida* under Cd stress (Xie et al., 2023). However, there have been few reports regarding the functions of lysophospholipids in response to Cd stress in higher plants.

In addition, we observed that with continuous Cd stimulation, the gene expression of *PLA* was initially downregulated at 36 h followed by upregulation at 72 h (**Figure 8**). PLA is critically important because it hydrolyzes the sn-1 and sn-2 positions of glycerophospholipids to produce lysophospholipids and free fatty acids (Hou et al., 2016). Previous studies have indicated a strong correlation between PLA and plant stress defense through its roles in the biosynthesis of JA and oxylipins, as well as the activation of downstream defense (Hou et al., 2016; Hu et al., 2023; Liang et al., 2023). Moreover, genes involved in α-linolenic acid metabolism, including FAD, *LOX*, *AOS*, *OPR*, and β-oxidation genes, exhibited significant upregulation at 72 h under Cd stress corresponding to significant increases in metabolites such as 13-HPOTrE, 9-HOTrE, and stearidonic acid (**Figure 8**). These findings suggest that phospholipase is activated in the plasma membrane promoting α-linolenic acid metabolism in *A. manihot* roots. The upregulation of the *LOX* and *AOS* genes, along with the increased levels of the crucial intermediate metabolite 13-HPOTrE, implies activation of the downstream JA pathway. Previous studies have indicated that under heavy metal stress, JA regulates antioxidant activity to enhance plant tolerance to heavy metal stress (Hewedy et al., 2023).

Interestingly, stearidonic acid, a nontraditional fatty acid, significantly increased at multiple time points. This may be attributed to the upregulation of *FAD* genes, which increases the conversion of α-linolenic acid to γ-linolenic and stearidonic acids (Lee et al., 2019). In *B. napus*, the transcript levels of the *FAD* genes were greater in the Cd-tolerant cultivar than in the sensitive cultivar under 250 μM Cd stress (Xu et al., 2019). Moreover, *FAD* genes have been extensively identified in diverse plant species; these genes are activated under various abiotic stress conditions and thereby augment plant stress resistance (Zhang et al., 2005; Yu et al., 2009; Xu et al., 2019). For instance, in tomatoes, *LeFAD3* overexpression significantly enhanced resistance to both salt and cold stress (Zhang et al., 2005; Yu et al., 2009). Therefore, we speculate that lipid metabolism maintains the stable cell membrane state in *A. manihot* under Cd stress and participates in the signaling processes involved in the plant stress response, thereby enhancing the Cd tolerance of plants.

### 4.5 Phenylpropanoid biosynthesis as an important metabolic pathway in response to Cd stress in *A. manihot*

Recent studies have highlighted the important role of phenylpropanoid biosynthesis in mitigating the adverse effects of Cd stress (Chen et al., 2020; Wang et al., 2022; Yu et al., 2023). In our studies, significant increases in the levels of trans-cinnamic acid and ferulic acid were detected at 72 h followed by pronounced increases in *p*-coumaric acid and sinapic acid levels at 7 days (**Figure 9**). Similarly, the accumulation of phenolic acids was detected in the roots of *Kandelia obovata* under Cd stress (Chen et al., 2020). On the one hand, enhancing phenolic acid biosynthesis in response to Cd exposure plays a crucial role in scavenging ROS due to their potent antioxidant activity (Sharma et al., 2019; Chen et al., 2020). On the other hand, phenolic acids are capable of chelating Cd or rendering Cd biologically unavailable, thereby mitigating its toxicity (Chen et al., 2020). It should be noted that the composition and concentration of phenolic acids differ among plants exposed to diverse heavy metal stresses (Anjitha et al., 2021). Such variations may be determinants of the specific defense strategies plants employ to cope with these environmental stresses. Consistent with these findings, structural genes involved in the synthesis of these compounds, including *PAL*, *C4H*, *COMT*, *F5H*, and *4CL*, were significantly upregulated at 72 h or 7 days (**Figure 9**). PAL is the first enzyme in the general phenylpropanoid pathway, catalyzing the nonoxidative elimination of ammonia to yield trans-cinnamic acid, which is then transformed into p-coumaric acid through a process catalyzed by C4H (Dong and Lin, 2021). Both trans-cinnamic acid and *p*-coumaric acid serve as precursors for a wide range of organic compounds and can be influenced by the metabolic efficiency of the phenylpropanoid, lignin, and flavonoid pathways (Chen et al., 2020; Dong and Lin, 2021).

In addition, significant reductions in the levels of three primary monolignols, namely, sinapyl alcohol, *p*-coumaryl alcohol, and coniferyl alcohol, were observed in the later stages of Cd stress (**Figure 9**). This phenomenon could be associated with the activation of REF1, which enzymatically converts coniferaldehyde and sinapaldehyde into sinapic acid and ferulic acid, respectively, thereby depleting the substrates necessary for monolignol synthesis (Nair et al., 2004). Interestingly, *REF1* was identified as a hub gene in the present study (**Figure 5C**). Nevertheless, the precise mechanism by which *REF1* influences the phenylpropanoid biosynthesis pathway in response to Cd stress requires further investigation. Additionally, an increase in the *POD* transcript level might also influence the monolignol content. The expression of the *GhPER8* gene, a ligninolytic peroxidase in tobacco leaves, significantly reduced the levels of coniferyl alcohol and sinapic acid, which are substrates for G-lignin and S-lignin biosynthesis, respectively (Gao et al., 2019). In summary, phenylpropanoid biosynthesis, through the production of phenolic acids and the modulation of lignin, plays a vital role in detoxifying *A. manihot* against Cd.

## 5 Conclusion

In this study, we conducted a comprehensive investigation of the physiology, transcriptome, and metabolome to elucidate the response mechanisms of *A. manihot* under Cd stress. At the physiological level, we identified the activation of the antioxidant system, including SOD, POD, and CAT, as a key mechanism for Cd detoxification in *A. manihot*. Analysis of the transcriptome and metabolome revealed dynamic effects of Cd stress on gene expression and metabolites in *A. manihot*. Integration of the physiology and transcriptome datasets allowed us to identify eight hub genes involved in processes, such as metal ion transport, ethylene response factors, and lignin biosynthesis, which are likely to play key roles in Cd stress responses. Moreover, the integration of transcriptome and metabolome datasets highlighted the critical role of phenylpropanoid biosynthesis, as well as lipid synthesis and metabolic pathways, in enhancing the tolerance of *A. manihot* to Cd. Overall, our study offers valuable insights into the mechanisms underlying the response of *A. manihot* to Cd toxicity. Moreover, these findings provide essential information for further exploration into the functional characterization of genes associated with Cd tolerance paving the way for future research aimed at improving Cd stress resilience in *A. manihot* and potentially other related plant species.

## Data availability statement

The data presented in the study are deposited in the NCBI repository, accession number PRJNA1078221.

## Author contributions

MW: Conceptualization, Data curation, Formal analysis, Methodology, Validation, Writing – original draft. QX: Data curation, Formal analysis, Validation, Software, Writing – review & editing. TT: Data curation, Software, Writing – review & editing. XL: Data curation, Software, Writing – review & editing. YP: Conceptualization, Formal analysis, Funding acquisition, Investigation, Methodology, Project administration, Resources, Supervision, Writing – review & editing.
