# Supplementary material for: Integrative physiological, transcriptomic, and metabolomic analysis of Abelmoschus manihot in response to Cd toxicity
Source: Front Plant Sci. 2024 Jun 10;15:1389207. doi: 10.3389/fpls.2024.1389207 (PMC11194374; doi:10.3389/fpls.2024.1389207)
Supplement: Supplementary file 2 [file DataSheet_1.pdf]

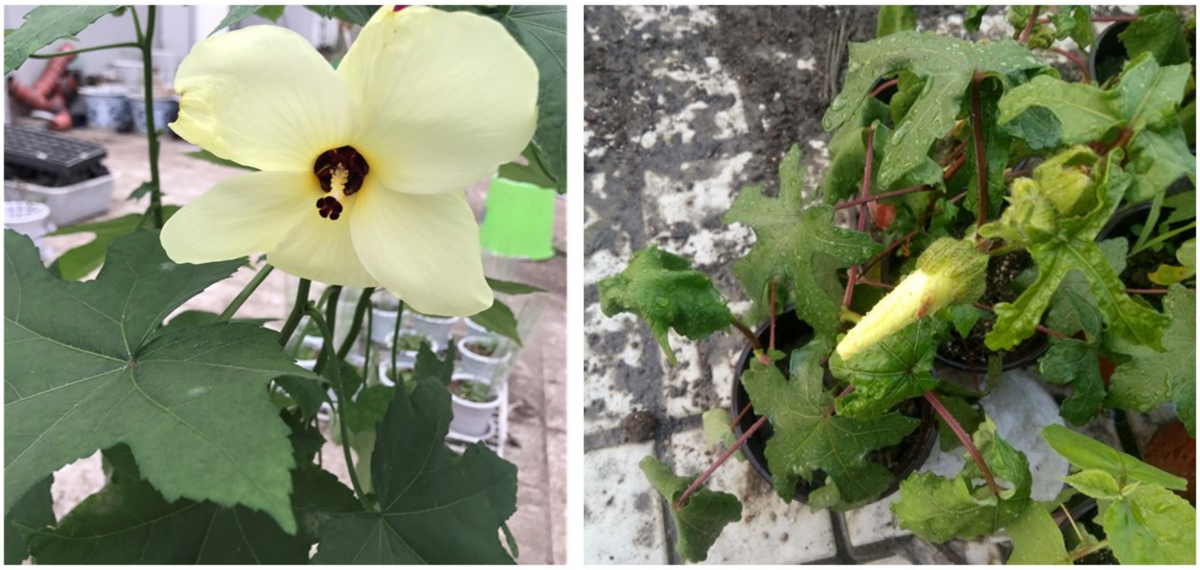

Figure S1 *Abelmoschus Manihot*

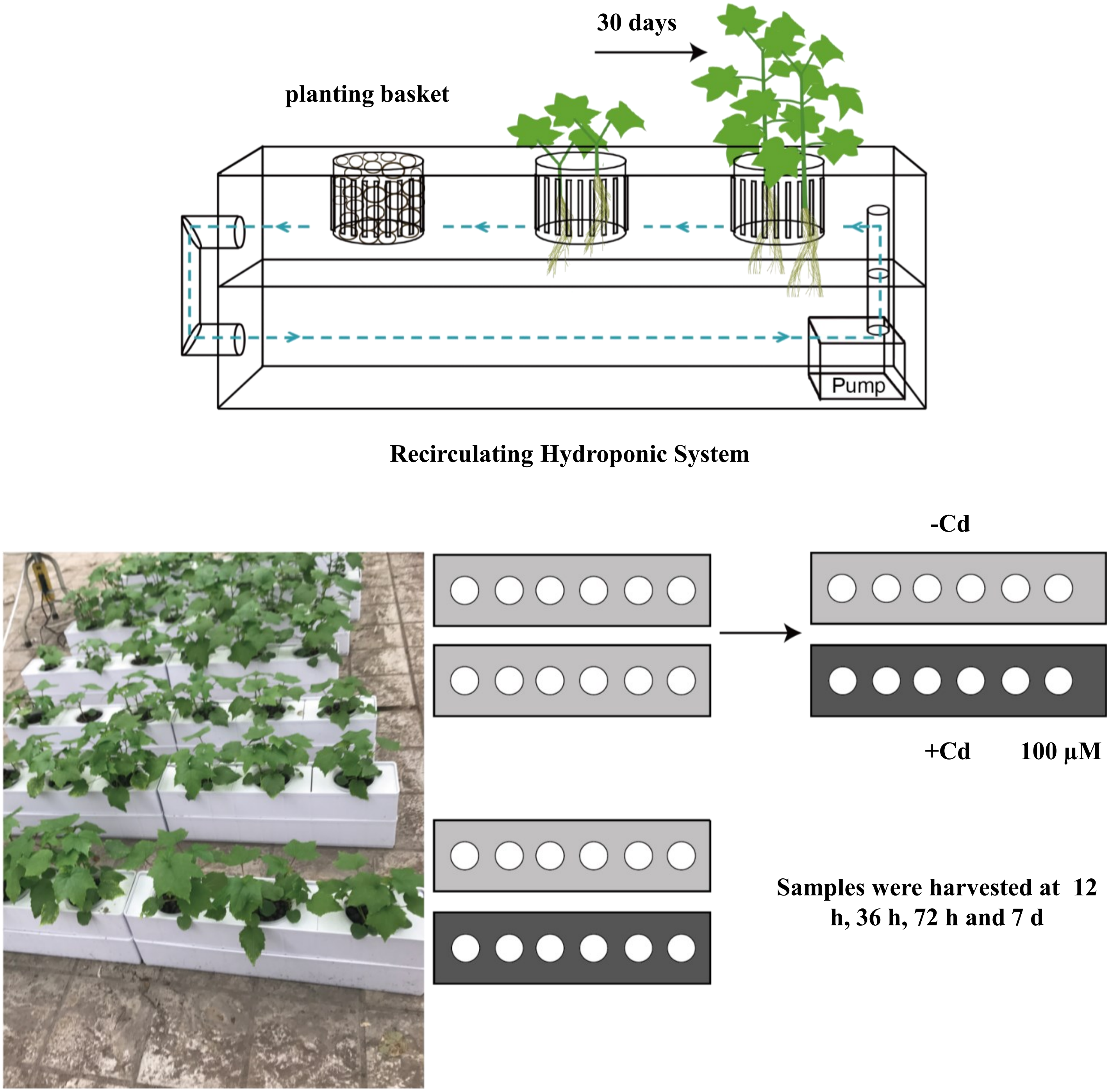

Figure S2 A hydroponic experiment of *A. manihot* under different Cd concentrations.

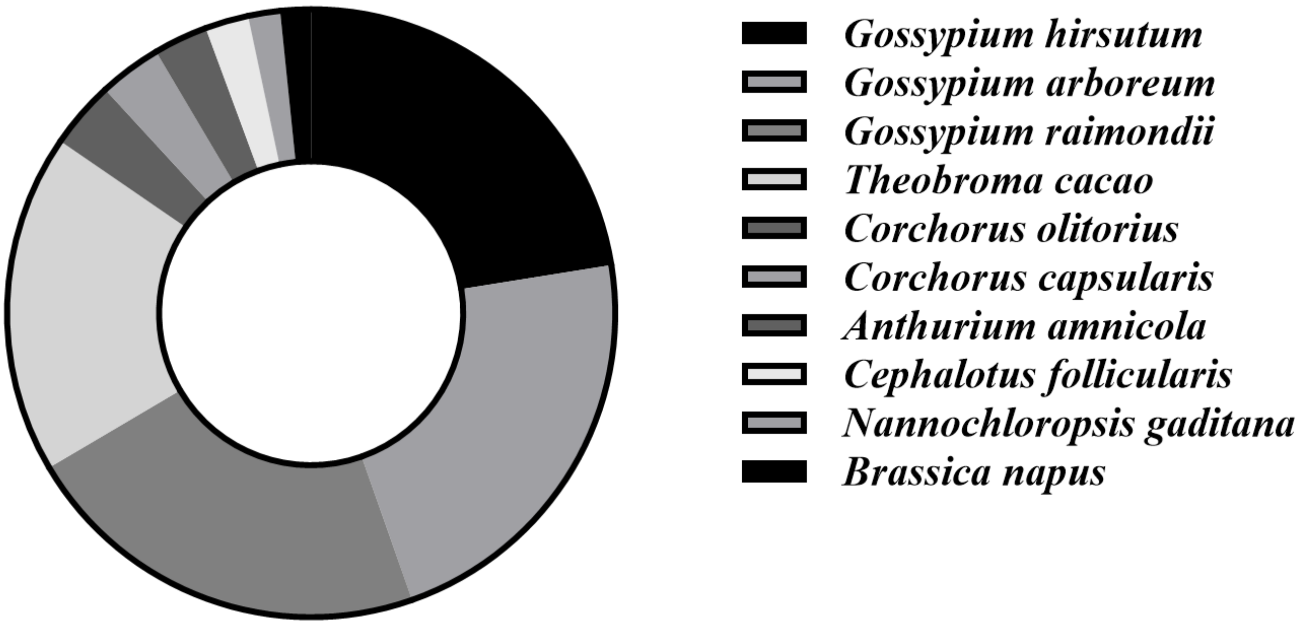

Figure S3 Species distribution of top 10 BLASTx hits against the NR database.

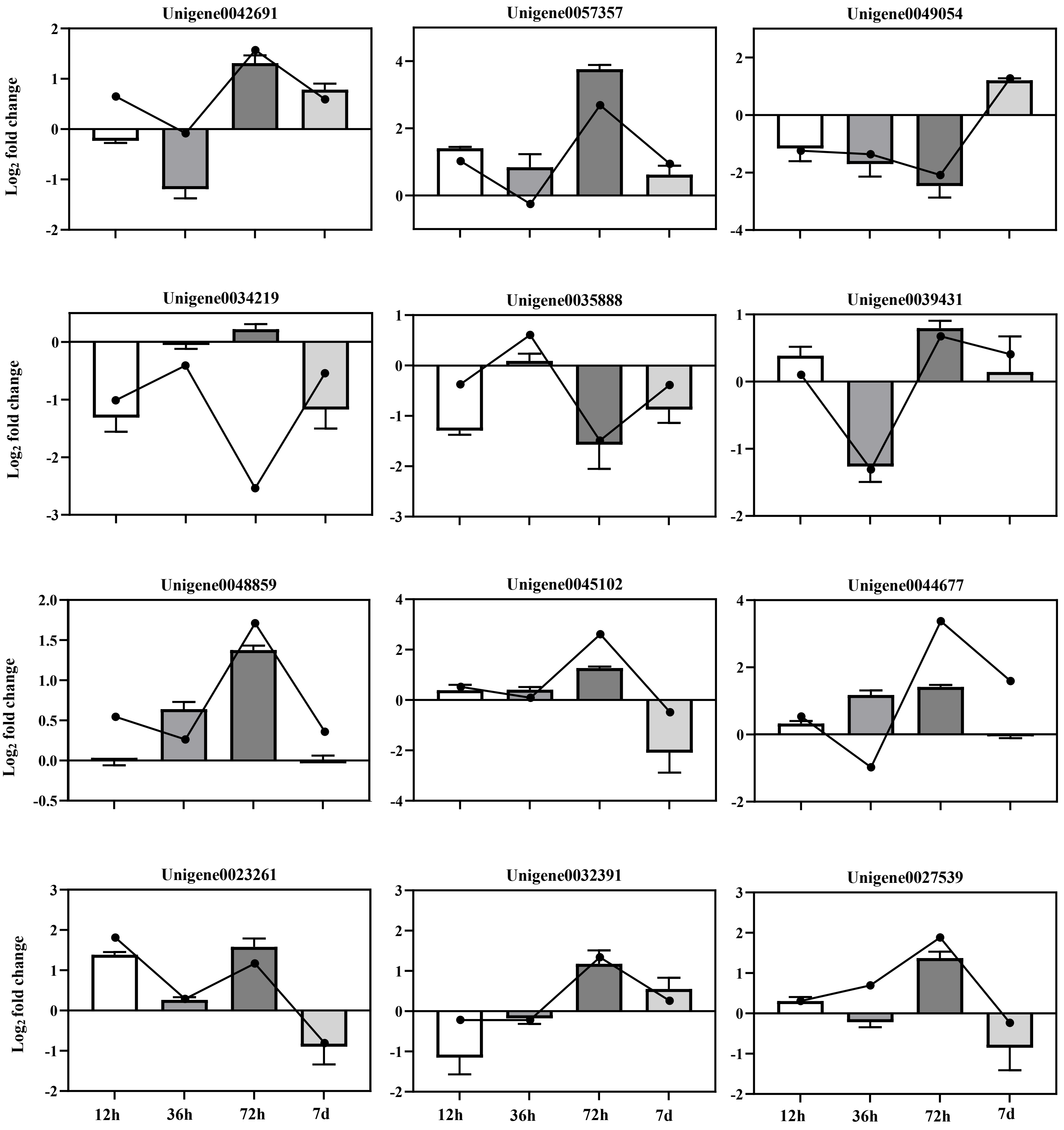

Figure S4 Validation of RNA-Seq results using qRT–PCR. Twelve DEGs were randomly selected for qRT–PCR assays. The bar charts indicate the qRT–PCR results, while the line charts represent the RNA-seq expression data.

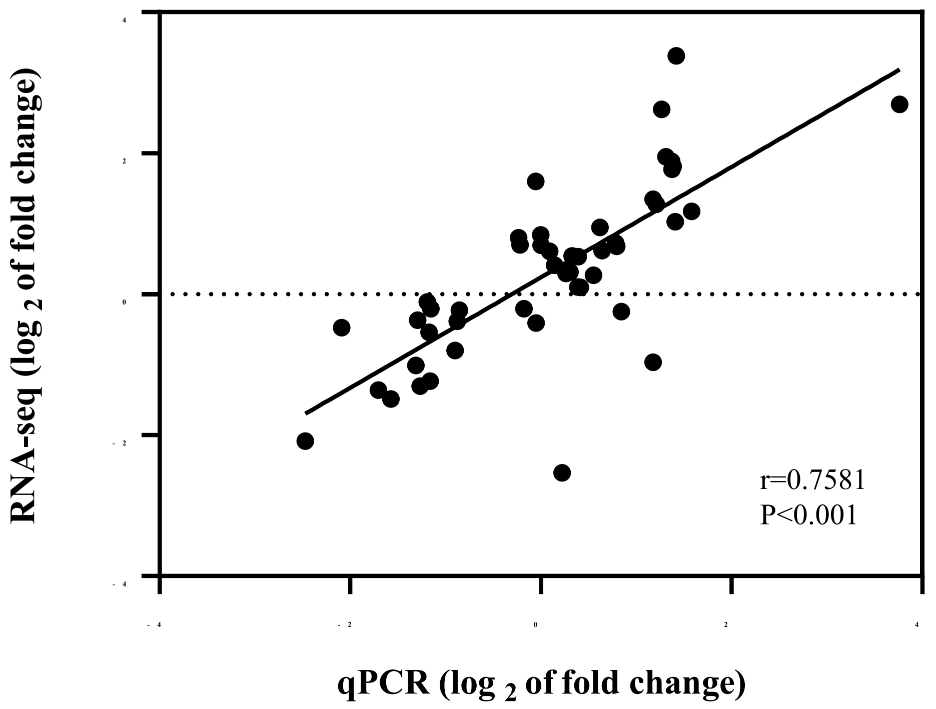

Figure S5 Correlation of gene expression levels between RNA-seq and qRT–PCR data for the selected genes.

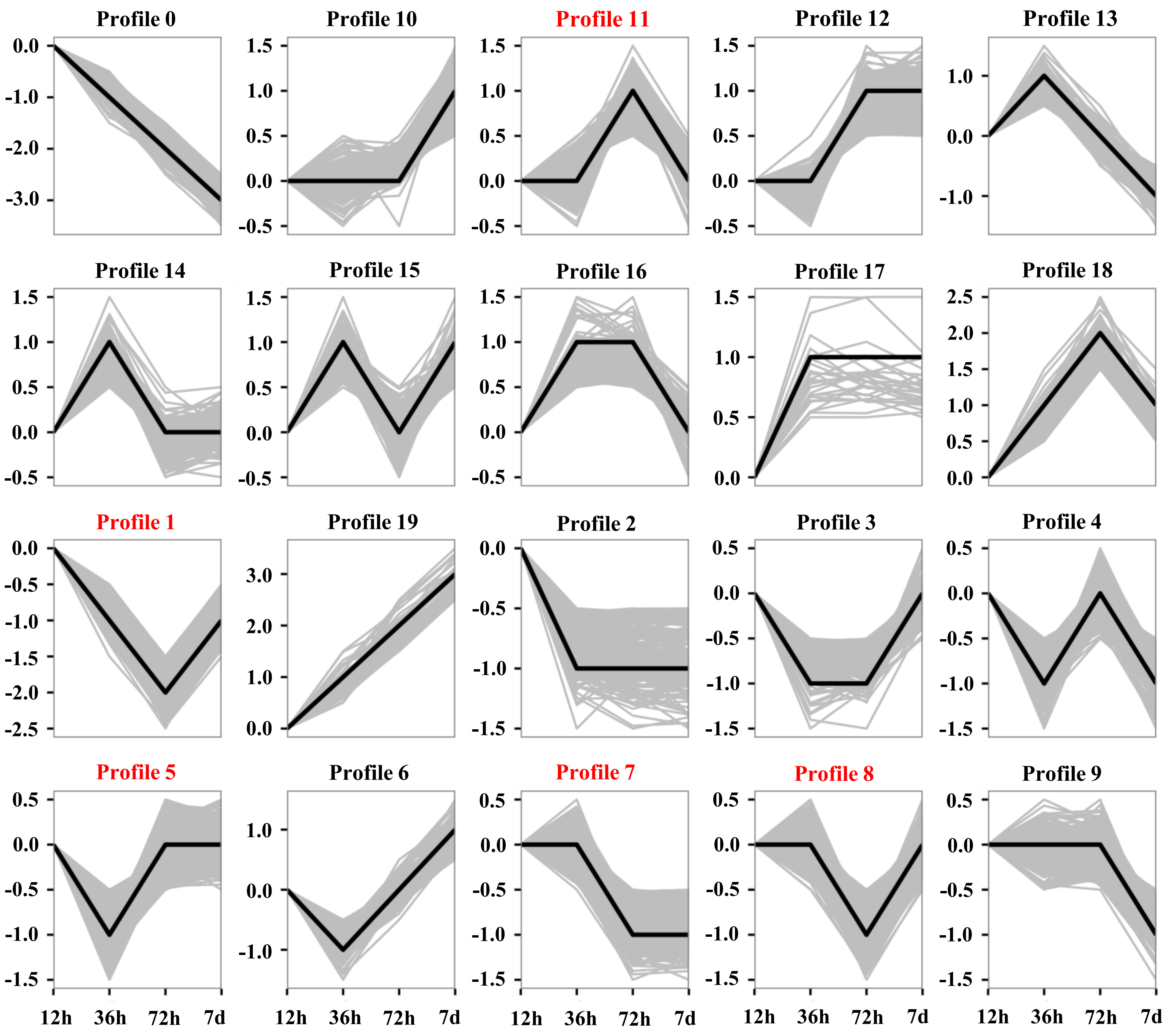

Figure S6 Short Time-Series Expression Miner (STEM) analysis of DEG expression profiles. The DEGs were mainly divided into 20 distinct temporal profiles using the 12 h samples as the baseline.

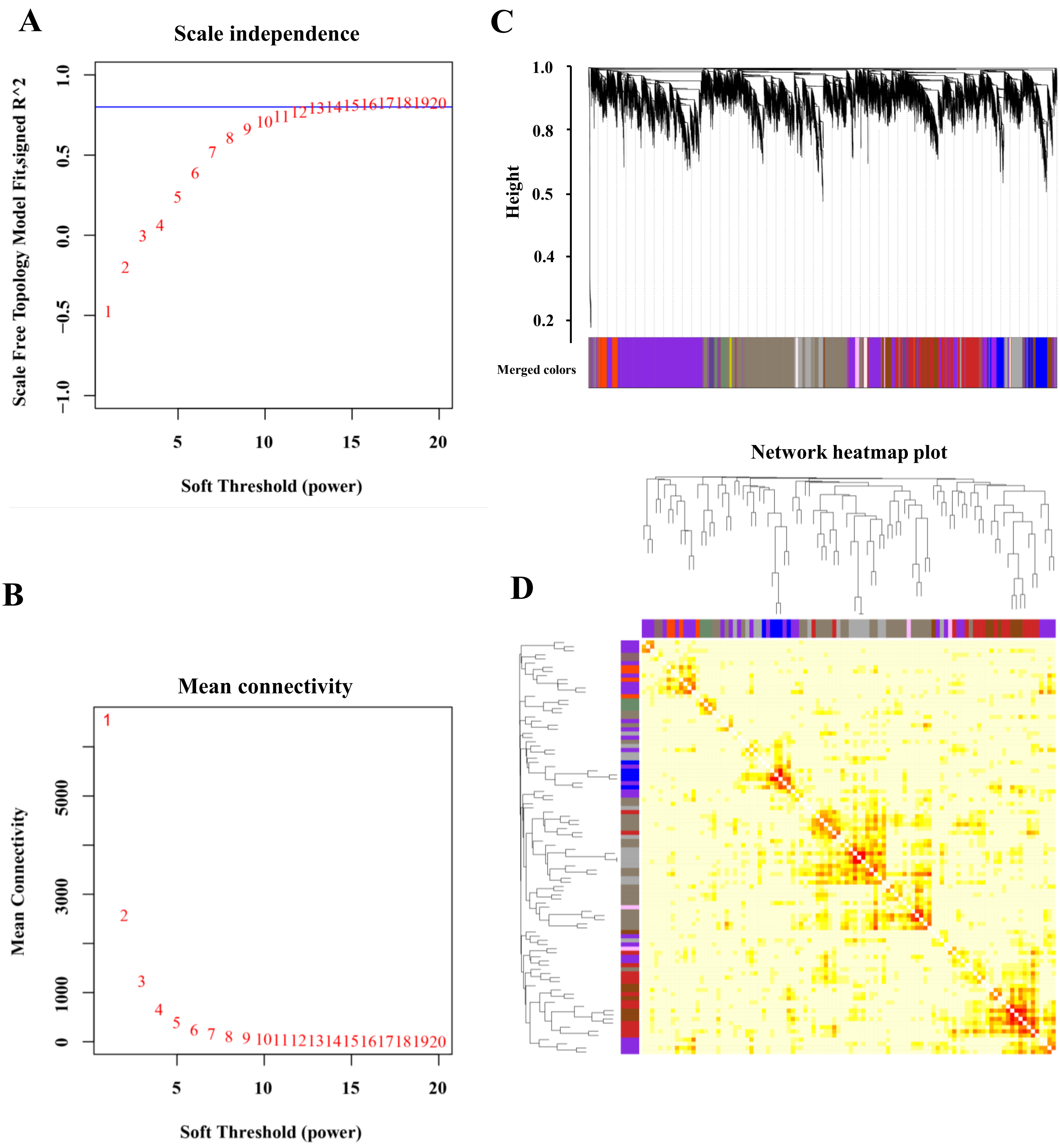

Figure S7 Construction of the coexpression network by WGCNA. (A) Determination of soft-thresholding power in WGCNA analysis (B) Analysis of the mean connectivity for various soft-threshold powers. (C) Hierarchical clustering tree showing 17 coexpression modules identified by WGCNA. Different modules are marked with different colors. (D) Heatmap of modular gene correlations. The tree represents a module (top and left), and the branch represents a gene. The darker the color of the dot (white → yellow → red), the stronger the connectivity between the two genes corresponding to the row and column.

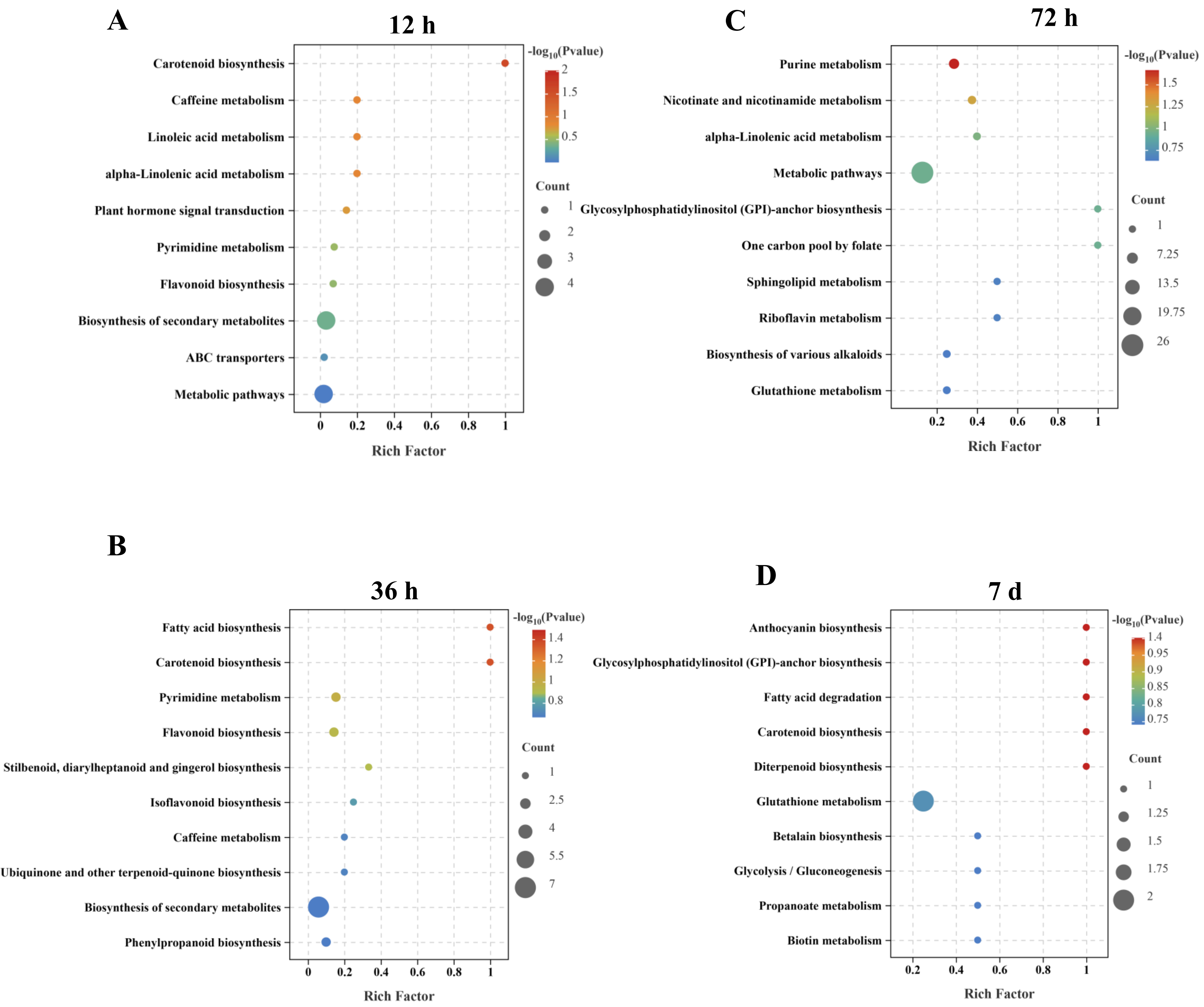

Figure S8 Top 10 enriched KEGG pathways for upregulated DEMs at (A) 12 h, (B) 36 h, (C)72 h, and (D) 7 d.
